# Rat Hair Metabolomics Analysis Reveals Perturbations of Unsaturated Fatty Acid Biosynthesis, Phenylalanine, and Arachidonic Acid Metabolism Pathways Are Associated with Amyloid-β-Induced Cognitive Deficits

**DOI:** 10.1007/s12035-023-03343-6

**Published:** 2023-04-25

**Authors:** Tian-Hoe Tan, Shih-Wen Li, Chih-Wei Chang, Yuan-Chih Chen, Yu-Hsuan Liu, Jui-Ti Ma, Ching-Ping Chang, Pao-Chi Liao

**Affiliations:** 1 Department of Emergency Medicine, Chi Mei Medical Center, Tainan, 710 Taiwan; 2 Department of Senior Services, Southern Taiwan University of Science and Technology, No.1, Nantai St., Yungkang Dist., Tainan, 710, Taiwan; 3 Department of Environmental and Occupational Health, College of Medicine, National Cheng Kung University, 138 Sheng-Li Road, Tainan, 70428 Taiwan; 4 Department of Medical Research, Chi Mei Medical Center, No. 901, Zhonghua Rd., Yongkang Dist., Tainan, 710 Taiwan; 5 Department of Food Safety/Hygiene and Risk Management, College of Medicine, National Cheng Kung University, Tainan, 701 Taiwan

**Keywords:** Targeted/untargeted metabolomics, Hair metabolism, Arachidonic acid, Unsaturated fatty acid, l-Phenylalanine

## Abstract

Hair is a noninvasive valuable biospecimen for the long-term assessment of endogenous metabolic disturbance. Whether the hair is suitable for identifying biomarkers of the Alzheimer’s disease (AD) process remains unknown. We aim to investigate the metabolism changes in hair after β-amyloid (Aβ_1-42_) exposure in rats using ultra-high-performance liquid chromatography-high-resolution mass spectrometry–based untargeted and targeted methods. Thirty-five days after Aβ_1-42_ induction, rats displayed significant cognitive deficits, and forty metabolites were changed, of which twenty belonged to three perturbed pathways: (1) phenylalanine metabolism and phenylalanine, tyrosine, and tryptophan biosynthesis—l-phenylalanine, phenylpyruvate, ortho-hydroxyphenylacetic acid, and phenyllactic acid are up-regulated; (2) arachidonic acid (ARA) metabolism—leukotriene B4 (LTB4), arachidonyl carnitine, and 5(S)-HPETE are upregulation, but ARA, 14,15-DiHETrE, 5(S)-HETE, and PGB2 are opposite; and (3) unsaturated fatty acid biosynthesis— eicosapentaenoic acid (EPA), docosahexaenoic acid (DHA), FA 18:3 + 1O, and FA 18:3 + 2O are downregulated. Linoleic acid metabolism belonging to the biosynthesis of unsaturated fatty acid includes the upregulation of 8-hydroxy-9,10-epoxystearic acid, 13-oxoODE, and FA 18:2 + 4O, and downregulation of 9(S)-HPODE and dihomo-γ-linolenic acid. In addition, cortisone and dehydroepiandrosterone belonging to steroid hormone biosynthesis are upregulated. These three perturbed metabolic pathways also correlate with cognitive impairment after Aβ_1-42_ stimulation. Furthermore, ARA, DHA, EPA, l-phenylalanine, and cortisone have been previously implicated in the cerebrospinal fluid of AD patients and show a similar changing trend in Aβ_1-42_ rats’ hair. These data suggest hair can be a useful biospecimen that well reflects the expression of non-polar molecules under Aβ_1-42_ stimulation, and the five metabolites have the potential to serve as novel AD biomarkers.

**Supplementary Information:**

The online version contains supplementary material available at 10.1007/s12035-023-03343-6.

## Introduction

Alzheimer’s disease (AD), a progressive neurodegenerative disease, is the most common form of dementia and results in memory loss, cognitive impairment, and behavioral changes [1]. More than 60% of dementia patients are diagnosed with AD. More than 24 million cases of AD have been diagnosed in recent years, and the prevalence continues to increase with the aging population [2]. The main features of AD are abnormal accumulation of amyloid-β (Aβ) peptide, which is derived from the sequential cleavage of amyloid precursor protein by β- and γ-secretases [3], and intracellular neurofibrillary tangles of hyperphosphorylated tau protein [4]. An increasing number of studies have shown that the oligomeric form of the Aβ peptide is a key initiator of neurotoxicity in AD pathogenesis [5]. The pathogenic events leading to AD are still controversial, but there is strong evidence indicating that oxidative stress and mitochondrial dysfunction play important roles in the neurodegenerative cascade [6]. Mitochondrial Ca^2+^ overload leads to increased reactive oxygen species (ROS) production in mitochondria [7], oxidative stress causes abnormal cellular calcium storage, and the inability to control oxidative stress or respond to metabolic disorders leads to a process linked to AD-causing gene mutations [8]. An AD diagnosis depends on cognitive function tests, medical history, positron emission tomography (PET) diagnosis, and detection of Aβ peptide or AD biomarkers in cerebrospinal fluid (CSF). However, PET analyses are expensive and time-consuming, and collecting CSF to detect Aβ peptide or AD biomarkers is difficult and invasive.

Metabolome profiling, focusing on metabolites and small molecules (< 1500 Da) implicated in most biological functions, has become an increasingly appreciated strategy for exploring the metabolic changes caused by diseases. Metabolomics provides excellent potential in the diagnosis and prognosis of neurodegenerative diseases [9]. However, the relationships between systemic abnormalities in metabolism and AD pathogenesis are unclear [10]. Studies have indicated that impaired amino acid metabolism is associated with AD pathogenesis, and altered amino acid signatures can be used as diagnostic biomarkers of AD [11]. In addition, a study has shown that the occurrence and progression of AD have a significant impact on central metabolites and related metabolic pathways, including energy-related metabolism (such as glycolysis, the tricarboxylic acid cycle), nitrogen metabolism (such as urea cycle and polyamine metabolism) disorders, fatty acid metabolism (for example, β-oxidation, and eicosanoid), neurotransmission (for example, serotonergic and dopaminergic neurotransmission), amino acid and nucleotide homeostasis, and others [12]. Therefore, the application of metabolomics has the potential to be used to identify biomarkers for AD diagnosis, discover novel therapeutic targets, and monitor therapeutic response and disease progression [10].

Untargeted approach based on ultra-high-performance liquid chromatography-high-resolution mass spectrometry (UHPLC-HRMS) is a valuable tool for metabolic analysis, and the breadth of its application has increased rapidly in the past decade. Targeted metabolomics measures characterized chemicals and biochemically annotates specific groups of metabolites. Several studies have shown that different potential AD biomarkers are found in different biospecimens, including CSF, blood, and urine [13]. However, metabolites in blood and urine have short stability periods with fluctuating totals [6]. Therefore, hair is a suitable sample because it is a valuable and noninvasive analytical specimen for the long-term and retrospective measurement of environmental substance exposure and endogenous metabolite perturbations and has been applied to the discovery of disease biomarkers [14–16]. The advantage of hair is its longer detection window than other biological specimens. In addition, hair as a bioanalytical sample has many advantages, including ease of sample collection, convenient transportation and storage, and ease of resampling. The effectiveness of hair matrix is becoming increasingly important in drug abuse, clinical settings, forensic toxicology, and metabolomics studies [14, 16]. Research on endogenous compounds in hair has focused on targeted approaches to specific compounds, such as testosterone, dihydrotestosterone, cholesterol, or cortisol [17]. Metabolomic information from the blood is incorporated into the hair during growth [14, 15]. Segmented hair analysis based on the hair growth rate can provide information about changes in metabolism over time [14]. Therefore, hair provides a wider detection window than other biological samples, such as blood or urine. In this study, we used an Aβ1-42-induced rat model of AD and HRMS-based untargeted and targeted metabolomics approaches to comprehensively profile potential AD biomarkers of hair metabolites.

## Materials and Methods

### Intracerebroventricular Injection of Aβ_1-42_

**Fig. 1:**
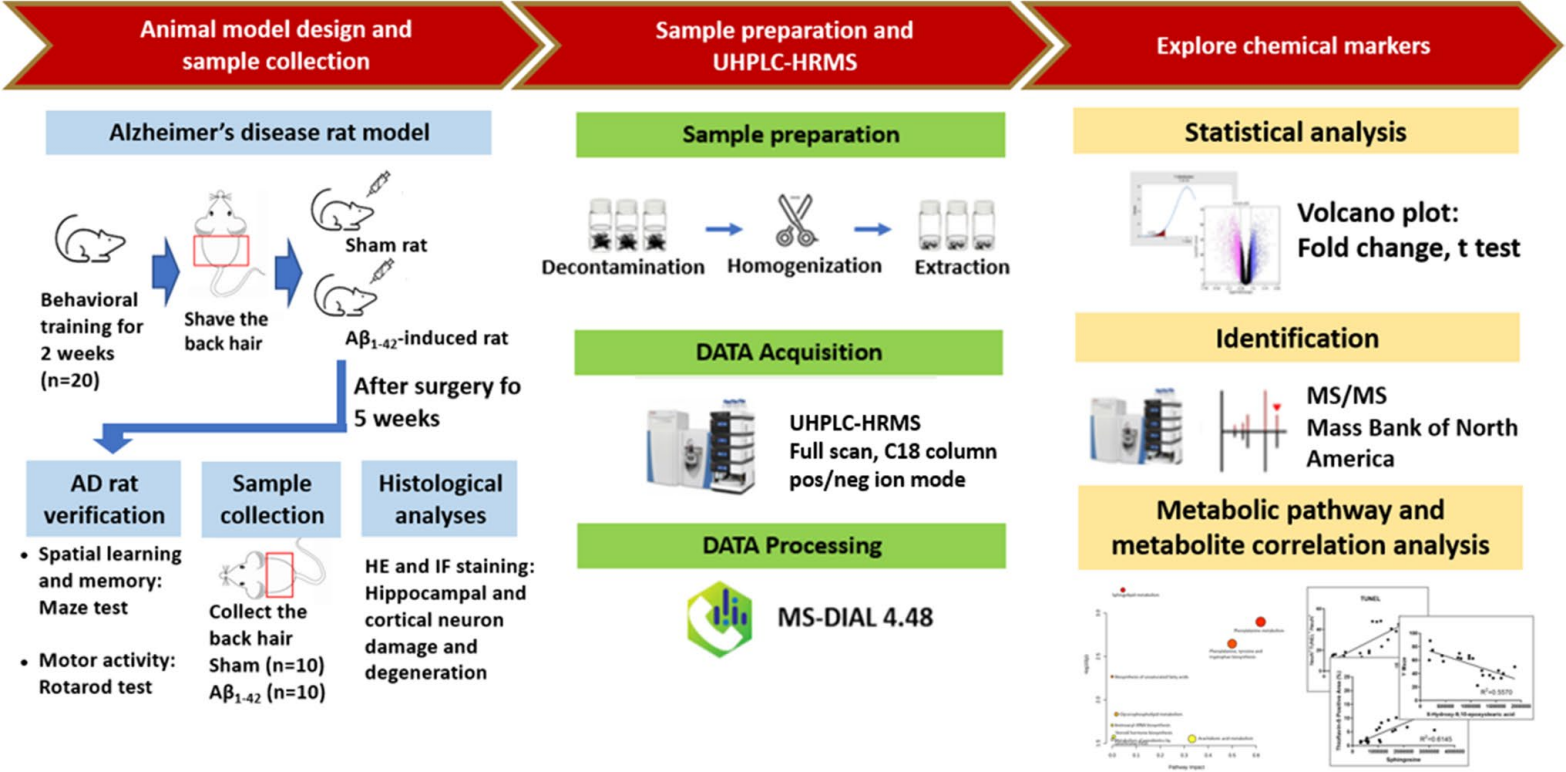
Experimental design. An overview workflow of the metabolomics analysis in AD. Hair samples from Aβ_1-42_-induced rats (*n* = 10 rats) and sham rats (*n* = 10 rats) were collected and subjected to untargeted metabolomics analysis. The AD rat model was verified based on motor activity, spatial learning and memory tests, and histological analyses. After hair sample decontamination, homogenization, and extraction, the hair extracts were analyzed with UHPLC-HRMS. The raw MS data were converted to peak lists using MS-DIAL for feature detection and alignment. The significantly altered features were identified by MS/MS analysis to explore chemical markers, and then the metabolic pathways were analyzed

The procedures for the Aβ_1-42_-induced rat model and infusion of Aβ_1-42_ into the bilateral cerebral ventricle were adopted from a previous study [18], and the Aβ_1-42_-induced rat model is verified by motor activity, spatial learning, and memory test. We purchased twenty 7-week-old male Wistar rats (RRID: RGD_13508588, weight of 250–350 g) from BioLASCO Co., Ltd., Taiwan (Taipei, Taiwan) and housed in an air-conditioned animal facility at 26 ± 0.5 °C under a 12-h light–dark cycle and water and food provided ad libitum at least 2 weeks before the start of experimentation. All behavioral tests were conducted between 10:00 a.m. and 04:00 p.m. The study protocols were approved by the Institute Animal Care and Use Committee of Chi Mei Medical Center, Tainan, Taiwan (IACUC approved no. 108120116). This study was not pre-registered. A statistical power analysis was performed for sample size estimation. Taking into account all parameters that yielded large effect sizes, we could ensure that a total sample size of 20 rats (*n* = 10 per group) could detect biologically relevant differences with a power of 80%. The animals (*n* = 20, the *n* means the number of rats) were randomly (by using https://www.randomizer.org/) allocated using a computer random group generator to one of 2 groups: Sham (*n* = 10) and Aβ_1-42_-induced rats (*n* = 10). All authors were aware of the group allocation at the different stages of the experiment. Rats were identified using a Stoelting™ Rat Ear Tag (Stoelting Co., Il, USA) for recognition and the testing order. Hairs were collected before and after the operation. Figure 1 displays the study design for the experiment. Animals found moribund or auto mutilating during the study were excluded and euthanized.

In the behavior training study, the inclusion/exclusion criteria of rats before surgery depends on the behavior tests, including Radial Maze, Y Maze, and Rotarod tests. During the training period, the rat had to be excluded if the rat stayed on the same arm for more than 2 min or the total completion time exceeded 10 min in the Radial Maze test, walked less than 10 steps within 8 min in the Y Maze test, or cannot run exceeding 1 min in the Rotarod test. All these 20 rats were included in surgery.

After 2 weeks of behavioral training, 9-week-old rats were anesthetized with a mixture of Zoletil (40 mg/kg; Virbac, Nice, France), xylazine hydrochloride (2 mg/kg; Balanzine, Health-Tech Pharmaceutical Co., Taipei, Taiwan), and atropine sulfate (1 mg/kg; Tai Yu Chemical & Pharmaceutical Co. Ltd., Hsinchu, Taiwan) by intramuscular injection; checked for complete anesthesia using a tail-pinch reflex test; and then placed in a Kopf stereotaxic apparatus (David Kopf Instruments, Tujunga, CA, USA). The rectal temperature of each rat was kept at 37 °C ± 0.5 °C using a homeothermic control unit (RightTemp® Temperature Monitor & Homeothermic Warming Control Module; Kent Scientific, Torrington, CT, USA). A middle sagittal incision was made in the scalp using standard sterilized procedures. Bilateral holes were drilled in the skull using a dental drill over the lateral ventricles (LVs). Bilateral intracerebroventricular (AP: − 0.8 mm; ML: ± 1.4 mm; DV: − 4.0 mm) injections were performed using a Hamilton microsyringe and a mini-pump (Hamilton, Reno, NV, USA). For induction of an AD model (*n* = 10), 20 μl of aggregated Aβ_1-42_ (1 μg/μl Sigma-Aldrich, USA, Cat.# SCP0038) at a rate of 0.5 μl/min was infused into bilateral LV (10 μl per ventricle). The syringe was removed 5 min after injection. The sham group (*n* = 10) received the same volume of the sterile vehicle solution. This day was designated as day 0. After surgery, the scalp was sutured, and sulfamethoxazole was sprinkled on the wound to prevent infection. In addition, penicillin (40,000U) will be injected intramuscularly into the gluteus once a day for 3 days [19]. Buprenorphine (0.05 mg/kg q12 h for 3 days, subcutaneously; Sigma-Aldrich, St. Louis, MO, USA) was used for postoperative analgesia. The surgery was performed under anesthesia to reduce the pain of the rat, and buprenorphine was given to relieve the pain after the operation. The rats were killed after complete anesthesia using Zoletil, xylazine hydrochloride, and atropine sulfate. This study was exploratory and no blinding was performed for experiments in this study. The behavioral tests were performed weekly from day 7 to day 35 post-surgery. On day 35 (the end-point of all experiments), the hair and brain samples were collected for histological and MS/MS analysis after the last behavior test.

### Behavioral Test

#### Radial Arm Maze Assay: Reference Memory and Working Memory Test

The maze consists of 8 arms extending radially from the central area. Before training, the rats were placed to explore the maze for 8 min and consume food freely. The rats were trained for 5 days to run to the end of the arm and consume baited food. The training trial continues until all 4 baits are used up. After adaptation, all rats were trained once a day for 3 days before Aβ_1-42_ injection and 35 days after Aβ_1-42_ injection. The number of working memory (short-term memory) errors (repeat entrance into an arm) and reference memory (long-term memory) errors (entrance into an arm that was never baited) were counted [20]. Latency was measured as the elapsed time from the start to the end of each trial. All measurements were averaged within each trial.

#### Y Maze Assay: Alternation, Novelty, and Memory Test

Short-term spatial recognition memory was assessed by analyzing spontaneous alternations in the Y maze [20]. Each rat was placed at the end of one arm, allowed to move freely in the maze for 8 min, and record a sequence of arm entries. Alternation was obtained when successive entries into three arms on overlapping triplet sets. The maximum number of possible spontaneous alternations was obtained as the total number of arms entered minus 2, and the percentage of alternation behavior was calculated as the ratio of actual to possible alternations × 100.

#### Rotarod Motor Coordination Test

Rotarod accelerating test was performed on each animal to assess motor coordination impairment [20]. The aim was to examine possible neuromuscular coordination deficits in Aβ_1-42_-injected rats. Each rat was placed on a rotator and subjected to accelerated testing. Place the rat on the rotarod (at the slowest speed, 4 rpm) for 1 min and accelerate to a maximum speed of 30 rpm at 3 min. On day 35 post-surgery, the rotational speed of the rod was subsequently accelerated to its maximum speed of 30 rpm. Measure the length of time the rat can grasp the rod. The test score is the average number of seconds the rat can hold the stick per trial.

### Histological Analyses

Molecular data come from the same animals after behavioral testing. Formalin-fixed brains were embedded in paraffin blocks. Serial sections through the hippocampus (− 2.8 mm anterior to bregma to − 4.3 mm anterior to bregma) and motor cortex (4.2 mm anterior to bregma to 1.2 mm anterior to bregma) were stained with hematoxylin and eosin for microscopy. Inspectors blinded to the experimental conditions assessed the degree of neuronal damage in each section. Damage scores were determined by two grading systems. In the first system, Honório et al. [21] determined a lesion score from 0 to 4 that indicates no pathological changes, lesions involving 25% of the field, and lesions involving 25 to 50%, lesions involving 50 to 75%, and lesions involving 75 to 100%, respectively. In another system, Liu et al. [22] used 0, 1, 2, and 3 for normal morphology, mild damage (edema, few pyknotic cells), moderate damage (structural disturbance, edema, moderate pyknotic cells, vacuolation, inflammatory cell infiltration), and severe damage (structural disturbance, edema, intense pyknotic cells, vacuolation, inflammatory cell infiltration), respectively. In our current study, we multiply these two scoring scores to arrive at the damage score [20].

### Thioflavin-S Stain

Amyloid plaques were stained with thioflavin-S. The sections were incubated in 0.25% potassium permanganate solution for 20 min, rinsed in distilled water, and then incubated in a bleach solution (2% oxalic acid and 1% potassium metabisulfite) for 2 min to deparaffinization and hydration. After rinsing with distilled water, sections were transferred to the blocking solution (1% sodium hydroxide and 0.9% hydrogen peroxide) for 20 min, incubated in 0.25% acidic acid for 5 s, washed with distilled water, and stained for 5 min in 50% ethanol 0.0125% Thioflavin-S was added (excitation/emission wavelength: 390/428 nm; #T1892, Sigma-Aldrich, MA, USA). Next, the sections were washed with 50% ethanol, placed in distilled water, and then covered with glass lids using a mounting medium [20].

### Triple Immunofluorescence Staining

NeuN^+^ cells were using Neu-N antibody (1:200, MAB377, Merck Millipore, Billerica, MA, USA) and Alexa Fluor 568–conjugated goat anti-mouse IgG (#A11004, Invitrogen) and then excited with 578 nm and observed through 603-nm emission. The sections were finally counterstained with 4′,6-diamidino-2 phenylindole (DAPI, 1:50,000, excitation/emission wavelengths: 358/461; #62,247, Thermo Fisher Scientific Inc., MA, USA) for identification of the nucleus.

For degenerative neuron detection, the brain tissue slides were immersed in a solution of 5% sodium hydroxide and 100% ethanol for 5 min, followed by immersion in 70% ethanol for 2 min, distilled water for 2 min, and 0.06% potassium permanganate solution for 10 min after secondary antibody incubation. The slides were then rinsed in distilled water for 2 min/2 times and incubated in a 0.0004% Fluoro-Jade B solution (#AG310, Millipore, MA, USA) that was made by adding 4 ml of a 0.01% stock solution of Fluoro-Jade B to 96 ml of 0.1% acetic acid. After 20 min incubation of the Fluoro-Jade B staining solution, the slides (green fluorescence with an excitation peak at 480 nm and emission peak at 525 nm) were thoroughly washed in distilled water, dehydrated, and coverslipped [20].

For neuronal apoptosis detection, the brain slides were treated with proteinase K (20 μg/ml) for 15 min at room temperature before the primary antibody incubation. Subsequently, an equilibration buffer was applied for 10 s, and the slides were immersed and incubated in working strength terminal deoxynucleotidyl transferase (TdT) enzyme solution at 37 °C for 1 h. Following incubation in stop/wash buffer for 10 min to terminate the reaction, the brain slides were then incubated for 30 min in working strength anti-digoxigenin conjugate at room temperature in the dark to visualize the DNA fragments. Proteinase K, equilibration buffer, and stop/wash buffer were all included in the terminal deoxyribonucleotide transferase–mediated dUTP nick end labeling (TUNEL) assay kit (excitation/emission wavelengths: 480/520 nm; #630,108, Takara Bio USA, Inc., CA, USA) [20]. TUNEL-positive neurons with condensed nuclei were identified as dead neurons. After a final wash with phosphate-buffered saline (PBS), the slides were mounted in glycerol gelatine mounting medium (#GG1-15 ML, Sigma-Aldrich, St. Louis, MO, USA) and viewed using an upright fluorescence microscope (Carl Zeiss Microscopy GmbH, Jena, Germany). Images were captured using a digital camera linked to a computer running Axioscope version 4 (Carl Zeiss). A pathologist counted the percentage of Fluoro-Jade + NeuN/DAPI and TUNEL + NeuN/DAPI triple-labeled cells in 6 fields per section in the hippocampus (× 400 magnification).

### Rat Hair Collection and Pretreatment

Thirty-five days after Aβ_1-42_ injection, the hair growing on the back was collected and then washed before the first step of hair pretreatment to remove external contamination. After hair collection, the rats were observed for the cognitive functions and behavioral tests, and then sacrificed for the histological analyses. The washing procedure was according to our previous study [14]. Twenty milligrams of rat hair was weighed in a glass tube and washed with acetone (3 mL) followed by deionized water (3 mL) in the ultrasonic bath for 2 min each. The washing solutions were then discarded, and hair samples were dried under nitrogen. Afterward, the dried hair samples were cut into snippets with a pair of scissors and subject to the extraction procedure. The hair sample (6 mg) was mixed with a 300 μL methanol:PBS 50:50 (v/v) solvent, and extracted under ultrasonic-associated extraction at 55 °C for 240 min in 37 kHz. Afterward, the extract was centrifuged at 15,000 × *g* for 15 min and the supernatants were collected. The hair extract was evaporated to dry by speed vac (EYELA, cue-2200), and the residue was reconstituted with 50% MeOH (30 µL) or stored at − 20 °C until analysis.

### UHPLC-HRMS Analysis

A UHPLC system coupled with a Q Exactive mass spectrometer (Thermo Fisher Scientific) was used for the sample analysis. The chromatographic separation was carried out by a Waters Acquity UHPLC BEH C18 column (2.1 mm × 100 mm, 1.7 μm). The mobile phase consisted of (A) 2% acetonitrile (ACN) in deionized water with 0.1% formic acid and (B) 100% ACN with 0.1% formic acid. The gradient conditions were as follows: 0–1 min, 2% B; 1–11 min, 2–99% B; 11–13 min, 99% B; 13–13.01 min, 99–2% B; 13.01–14 min, 2% B. The flow rate was set at 0.35 mL/min. The column temperature was kept at 40 °C and the injection volume was 5 μL. The electrospray ionization was operated in both positive and negative modes. The mass range of m/z 100–1000 was recorded at the resolution of 70,000 in MS1. The MS/MS operating in data-dependent acquisition mode with a targeted mass list was adopted for the fragmentation using higher-energy collisional dissociation with normalized collision energies at 10, 20, 50, and 100%, respectively.

### Data Processing and Statistical Analysis

There were no outliers excluded from the statistical analysis. We performed two-way ANOVA with Tukey’s multiple comparison tests to analyze behavioral performance using GraphPad Prism 7.01 (GraphPad Software Inc., CA, USA). The behavior data were expressed as mean ± standard error of the mean (SD). Parameters of histological scores and the immunofluorescence staining data with non-normal distribution were analyzed by Mann–Whitney *U* Test. The correlation coefficients between the abundance of metabolites and behavior, thioflavin-S stain, Fluoro-Jade + NeuN/DAPI double-labeled stain, and TUNEL + NeuN/DAPI double-labeled staining were analyzed by linear regression. *P*-values < 0.05 were considered statistically significant.

The MS data were processed using MS-DIAL [23] for compound detection, alignment, and identification. During the alignment, tolerance for retention time was set at 0.2 min and for mass error it was set at 0.01 Da. Peak tables containing accurate m/z, retention times, and peak abundances were exported. The features of signal/noise (S/N) < 3 were considered the absence of peaks and hence filtered. Before performing a univariate analysis to discover the differential features, each raw abundance value was normalized by dividing it by the sum of the raw abundance values of all peaks in the corresponding sample using Microsoft Excel [24]. Differential features were evaluated with volcano plots based on a fold change | log_2_ (fold-change) |≥ 1 and *P*-value of two-tail unpaired Student *t*-tests ≤ 0.05. The identification of biomarker candidates was performed based on the accurate mass and MS/MS fragmentation spectra matching by MS-DIAL with MassBank of North America (MoNA) and MS-Finder, PubChem, Human Metabolome Database, LIPID MAPS, and mzCloud database as the backend knowledge base. To discover the alterations in metabolic pathways associated with the Aβ_1-42_-induced rats compared with sham rats, we used the online software MetaboAnalyst 5.0 (www.metaboanalyst.ca) and SMPDB (The Small Molecule Pathway Database) [25] to perform pathway analysis.

## Results

### Experimental Design

**Fig. 2:**
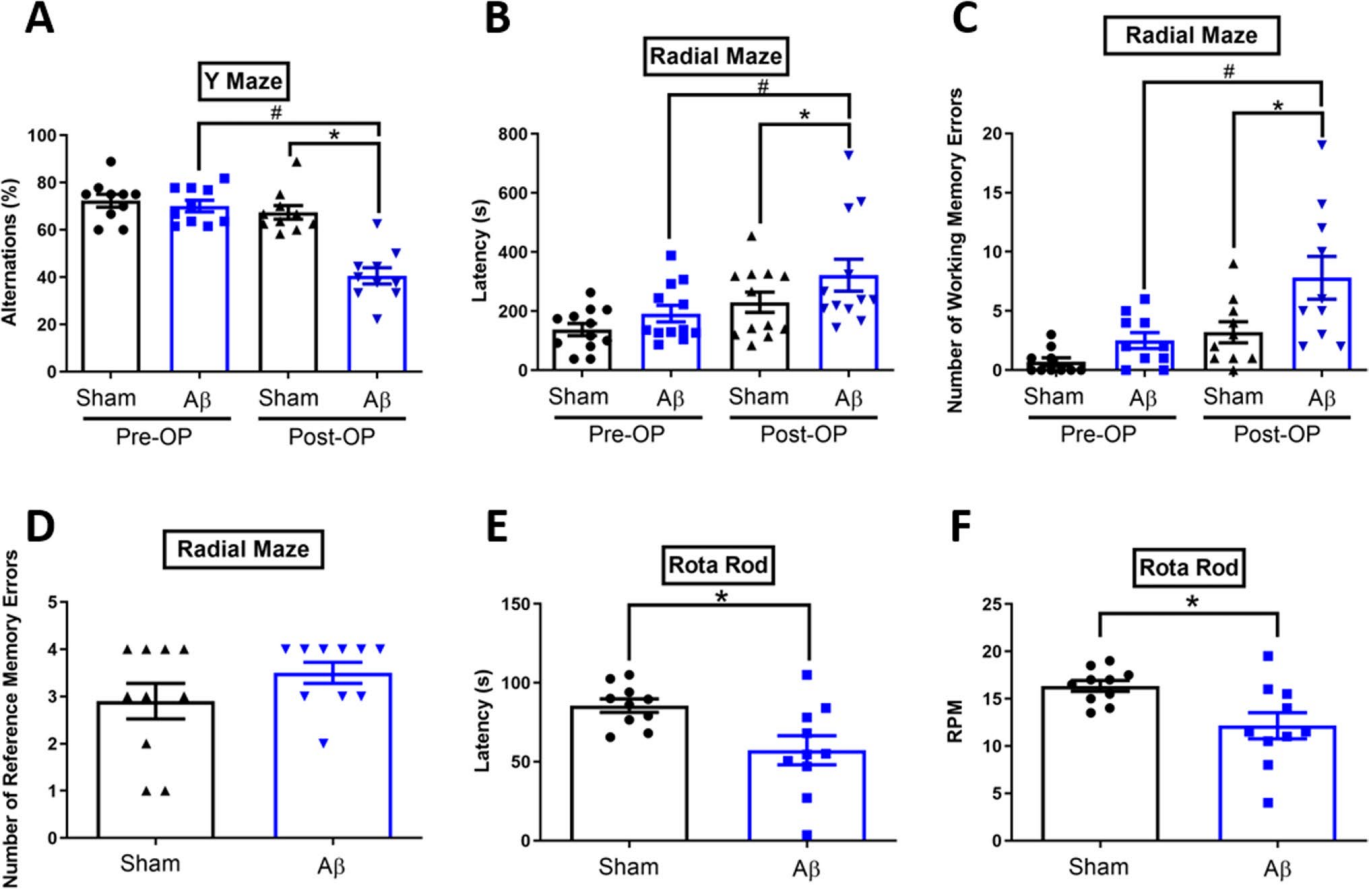
Learning and memory performance and motor activity for sham and Aβ_1-42_-induced rats. The spontaneous alternation in the **A** Y-maze, **B** the latency to elapse, **C** numbers of working memory errors, and **D** numbers of reference memory errors in the radial maze tests, and **E** the latency to fall off the rotarod and **F** the maximum speed reached during the test in the rotarod tests were presented. Data are present mean ± SD. The number of animals used was *n* = 10 for each experimental group. *p < 0.05 versus the Sham group. ^#^*p* < 0.05 versus the Pre-OP Aβ  group. Pre-OP, preoperation; Post-OP, postoperation

**Fig. 3:**
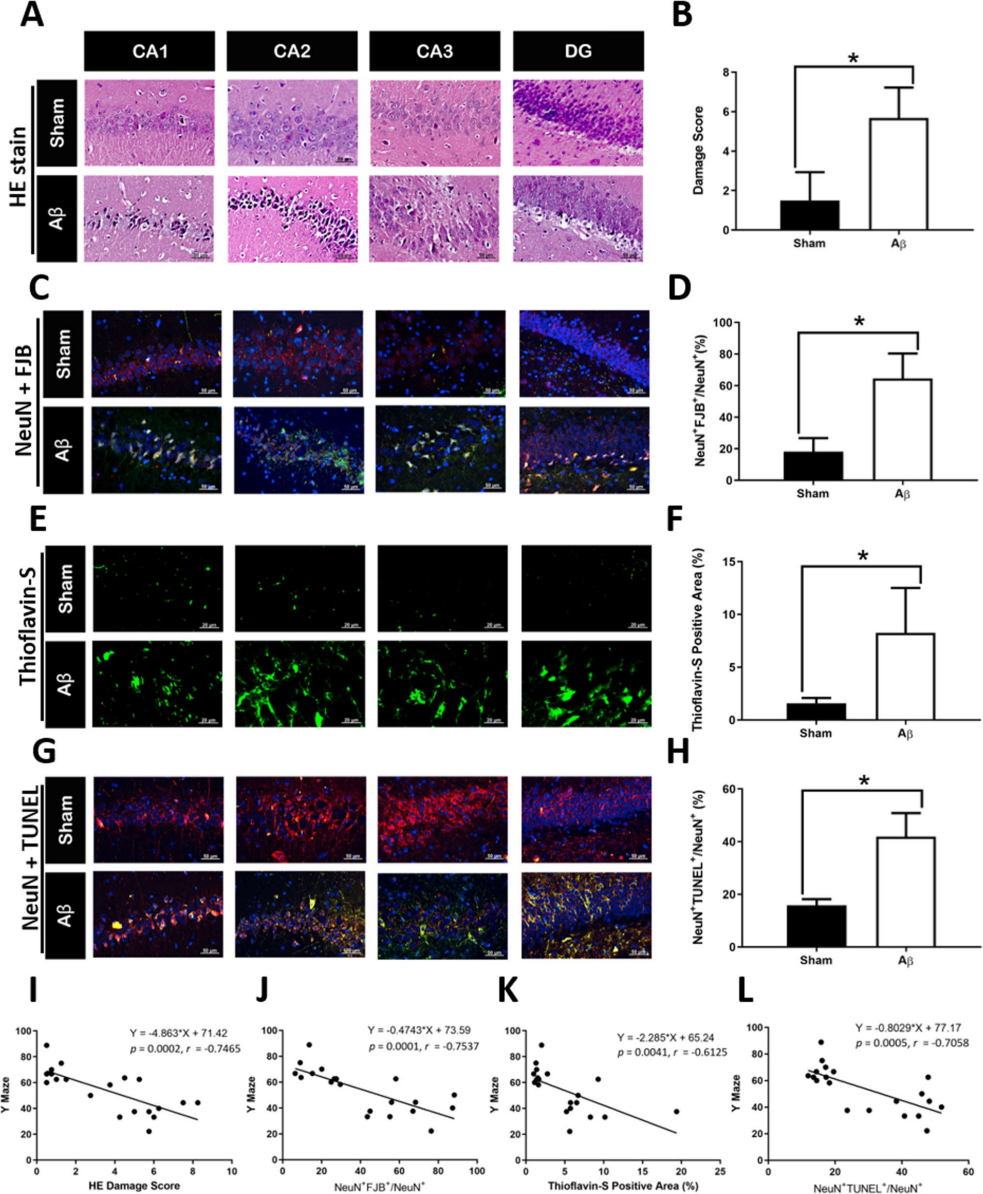
Histological analysis in Aβ_1-42_-induced rats and sham rats and correlation analysis between behavioral test and histological effects. Histology of hippocampus from Aβ_1-42_-induced and sham rats using HE staining (**A**, **B**), NeuN + Fluoro-Jade B staining (**C**, **D**), thioflavin-S staining (**E**, **F**), and NeuN + TUNEL staining (**G**, **H**). Correlation analysis between Y maze test and (**I**) HE/ (**J**) NeuN + Fluoro-Jade B/ (**K**) Thioflavin-S/ (**L**) NeuN + TUNEL staining. The number of animals used was *n* = 10 for each experimental group

**Fig. 4:**
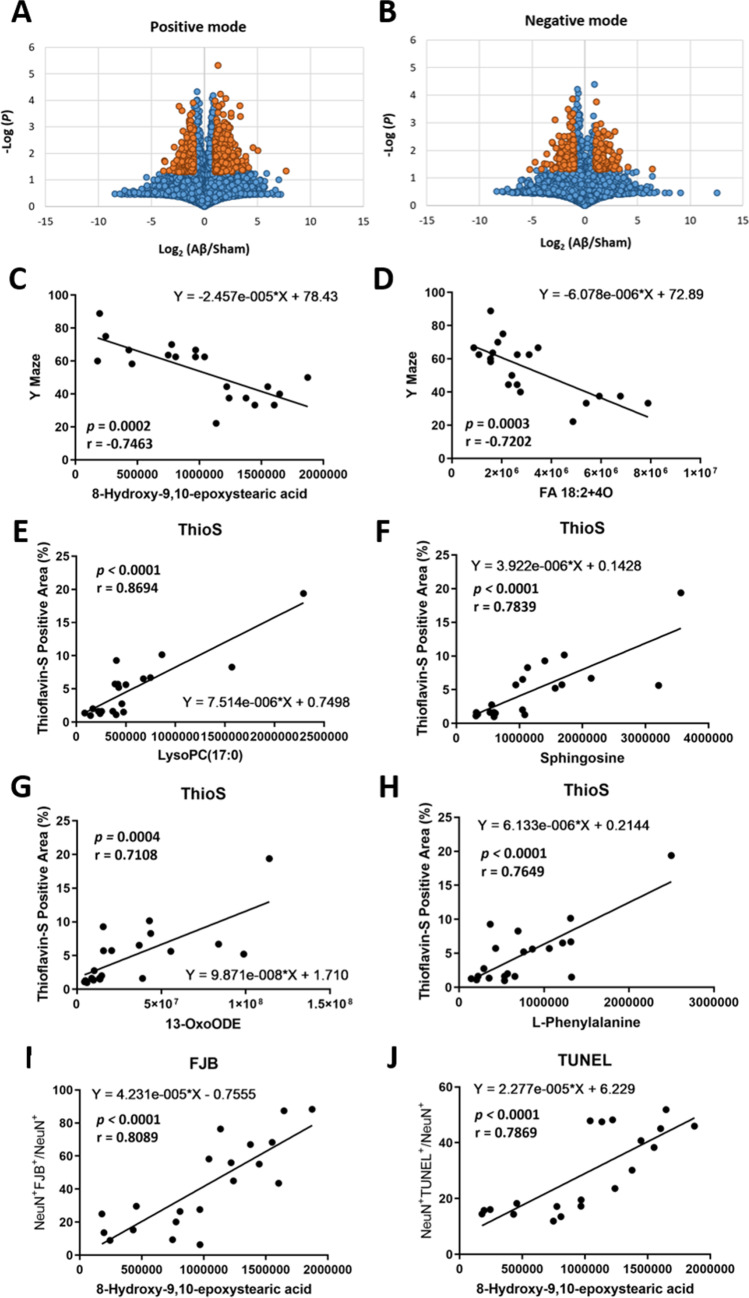
Differential metabolites and the correlation between metabolites and histological analysis and behavioral test in Aβ_1-42_-induced rat. Volcano plot analysis illustrated the identified features between Aβ_1-42_-induced and sham rats in **A** positive and **B** negative mode. The orange spot represents the features displayed with larger magnitude fold changes (*x*-axis, |log_2_(Aβ_1-42_/Sham) |≥ 1.0) and statistical significance difference (*y*-axis, -log *P*-value ≥ 1.33). The correlations between **C** Y maze and 8-hydroxy-9,10-epoxystearic acid, **D** Y maze and FA 18:2 + 4O, **E** Thio-S staining and LysoPC(17:0), **F** Thio-S staining and sphingosine, **G** Thio-S staining and 13-OxoODE, **H** Thio-S staining and l-phenylalanine, **I** FJB staining and 8-hydroxy-9,10-epoxystearic acid, and **J** TUNEL staining and 8-hydroxy-9,10-epoxystearic acid. The number of animals used was *n* = 10 for each experimental group

**Fig. 5:**
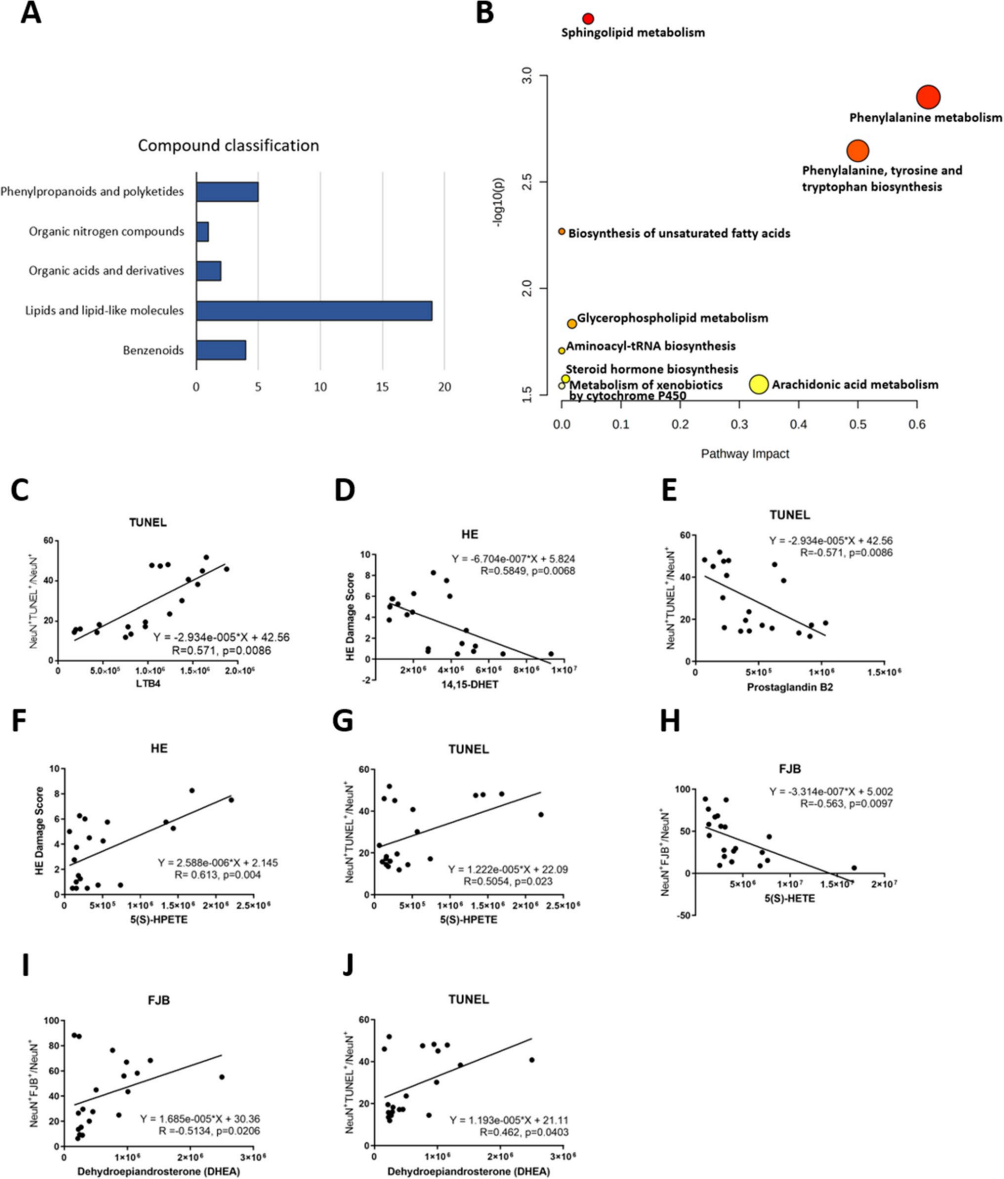
Classification, pathway analysis, and correlations of the differential hair metabolites between Aβ_1-42_-induced and sham rats. **A** Thirty-three differential hair metabolites were classified. **B** Pathway analysis based on differential metabolites using online software MetaboAnalyst (www.metaboanalyst.ca). The correlations between **C** TUNEL staining and LTB4, **D** HE staining and 14,15-DHET, **E** TUNEL staining and PGB2, **F** HE staining and 5(S)-HPETE, **G** TUNEL staining and 5(S)-HPETE, **H** FJB staining and 5(S)-HETE, **I** FJB staining and DHEA, and **J** TUNEL staining and DHEA. The number of animals used was *n* = 10 for each experimental group

In this study, we developed an experimental workflow to compare the metabolic differences between Aβ_1-42_-induced rats and sham rats using UHPLC-HRMS (Fig. 1). For the animal design and sample collection, the hair on the back of the rats was shaved after 5 weeks of Aβ_1-42_ intracerebroventricular injection. The Aβ_1-42_-induced AD rat model was verified based on motor activity, spatial learning and memory tests (Fig. 2), and histological analyses of the brain tissue (Fig. 3). Rat hairs were collected, prepared, extracted, and analyzed by UHPLC-HRMS. The significant peak abundance changes, the correlation between metabolite candidates and behavioral test/histological analyses, compound classification, and metabolic pathways are shown in Figs. 4 and 5. The metabolites of the metabolic pathways were verified using targeted UHPLC-HRMS.

### Bilateral i.c.v. Aβ_1-42_ Injection Induces Motor and Cognitive Deficits Accompanied by Histological Changes in Rats

To determine whether i.c.v. administration of Aβ_1-42_ affects learning and memory and motor coordination of rats, the Y-maze, radial maze, and rotarod tests were performed. Compared to the Sham group, the Aβ_1-42_-treated rats exhibited the following: decreased spontaneous alternations percentage within the Y-maze task (two-way ANOVA factoring time and group, time difference *F* (1, 36) = 38.46, *P* < 0.0001; group difference *F* (1, 36) = 25.94, *P* < 0.0001; time-group interaction *F* (1, 36) = 18.54, *P* = 0.0001; Fig. 2A), increased time taken to consume all four bits (two-way ANOVA with time and group as factors, time difference *F* (1, 36) = 9.780, *P* = 0.0035; group difference *F* (1, 36) = 6.859, *P* = 0.0128; time-group interaction *F* (1, 36) = 2.270, *P* = 0.1407; Fig. 2B), and working memory errors (two-way ANOVA with time and group as factors, time difference *F* (1, 36) = 13.60, *P* = 0.0007; group difference *F* (1, 36) = 10.26, *P* = 0.0028; time-group interaction *F* (1, 36) = 2.119, *P* = 0.1541; Fig. 2C), but insignificance of reference memory errors (Mann–Whitney *U* test, *P* = 0.2987) within the radial maze task (Fig. 2D), and decreased latency (Mann–Whitney *U* test, *P* = 0.0138; Fig. 2E) and speed of motor performance (Mann–Whitney *U* test, *P* = 0.0175; Fig. 2F) within the rotarod task.

The rats were sacrificed for histological analysis after hair collection and behavioral test. Rats with intracerebroventricularly microinjected of Aβ_1-42_ exhibited neuronal degeneration (shown by hematoxylin–eosin [HE] staining [Mann–Whitney *U* test, *P* < 0.0001; Fig. 3A and B] and NeuN with Fluoro-Jade B [FJB] staining [Mann–Whitney *U* test, *P* < 0.0001; Fig. 3C and D]), Aβ accumulation (evidenced by thioflavin-S [Thio-S] staining [Mann–Whitney *U* test, *P* < 0.0001; Fig. 3E and F]), and apoptosis (evidenced by NeuN and TUNEL staining [Mann–Whitney *U* test, *P* < 0.0001; Fig. 3G and H]). The correlations between Y maze and HE/FJB/thioflavin-S/TUNEL stains are shown in Fig. 3I–L. The Aβ_1-42_-induced rats displayed high score of neuronal degeneration, Aβ accumulation, and apoptosis compared with sham rats. In addition, the behavioral test of Y maze was high correlation (|*R* value|> 0.7) with HE, FJB, and TUNEL staining (Fig. 3I, J, and L). The correlation between histopathological staining and neurobehavioral tests are summarized in Supplementary Table 1.

### Untargeted Metabolomics Reveals Dysregulations in Phenylalanine, Phenylalanine, Tyrosine and Tryptophan Biosynthesis, and Arachidonic Acid Metabolism Pathways After Aβ_1-42_ Induction

The hair extracts were analyzed using UHPLC-HRMS in both positive and negative ion modes. A total of 16,536 peaks in positive ion mode and 9523 peaks in negative ion mode were aligned in both AD and sham rats. The significant peak abundance changes between Aβ_1-42_-induced rats and sham rats (| log_2_ (Aβ_1-42_ / sham) |≥ 1 and *P*-value ≤ 0.05) are shown in the volcano plots (Fig. 4A and B, the dark blue spots). In positive ion mode, 409 peaks were upregulated in AD rats, whereas 222 peaks were downregulated. In addition, 167 peaks were upregulated, and 160 peaks were downregulated in negative ion mode. A total of 958 peaks were subjected to MS/MS for chemical structure identification. Thirty-three metabolites were identified through the MoNA database within MS-DIAL, including 20 metabolites and 13 xenobiotic exposure perturbations. Eighteen metabolites were identified in positive ion mode, and fifteen metabolites were identified in negative ion mode (Table 1). The correlations between these 23 metabolites and behavioral test/histological analysis were analyzed by linear regression (Supplemental Table 2). 8-Hydroxy-9,10-epoxystearic acid, FA 18:2 + 4O, lysoPC(17:0), sphingosine, 13-OxoODE, and l-phenylalanine showed significantly high correlational *R* values (|*R*|> 0.7) with Y maze, Thio-S staining, FJB staining, and TUNEL staining, respectively (Fig. 4C–J and Supplemental Table 2).

**Table 1:** Differential hair metabolites accountable for the discrimination between Aβ_1-42_-induced and sham rats

Compound name	RT (min)	m/z	Ion mode	Metabolites/xenobiotic exposure	PubChem CID	HMDB	Formula	Superclass	FC (Aβ/Sham)	*P* value
4-Hydroxyquinoline	6.46	144.0436	[M − H] −	Xenobiotic exposure	69,141	HMDB0246466	C9H7NO	Organoheterocyclic compounds	0.46	0.0306
3-Methylsalicylic acid	5.71	151.0383	[M − H] −	Xenobiotic exposure	6738	HMDB0002390	C8H8O3	Benzenoids	1.99	0.0142
Ortho-Hydroxyphenylacetic acid	5.34	151.0390	[M − H] −	Metabolites	11,970	HMDB0000669	C8H8O3	Benzenoids	2.45	0.0021
Pimelic acid	1.46	159.0658	[M − H] −	Xenobiotic exposure	385	HMDB0000857	C7H12O4	Lipids and lipid-like molecules	2.91	0.0256
2-Hydroxycinnamic acid	4.32	163.0384	[M − H] −	Xenobiotic exposure	637,540	HMDB0002641	C9H8O3	Phenylpropanoids and polyketides	1.88	0.0329
Phenylpyruvic acid	7.49	163.0396	[M − H] −	Metabolites	997	HMDB0000205	C9H8O3	Benzenoids	2.54	0.0273
3-(3-Hydroxyphenyl)propanoic acid	4.01	165.0547	[M − H] −	Xenobiotic exposure	91	HMDB0000375	C9H10O3	Phenylpropanoids and polyketides	2.30	0.0393
Phenyllactic acid (Phenylpyruvate)	6.39	165.1274	[M − H] −	Metabolites	3848	HMDB0000779	C9H10O3	Phenylpropanoids and polyketides	3.30	0.0089
l-Phenylalanine	2.27	166.0860	[M + H] +	Metabolites	6140	HMDB0000159	C9H11NO2	Organic acids and derivatives	2.17	0.0224
(2-Oxo-2,3-dihydro-1H-indol-3-yl)acetic acid	3.75	190.0493	[M − H] −	Xenobiotic exposure	3,080,590	HMDB0035514	C10H9NO3	Organoheterocyclic compounds	0.44	0.0477
Sebacic acid	5.25	201.1129	[M − H] −	Metabolites	5192	HMDB0000792	C10H18O4	Lipids and lipid-like molecules	2.15	0.0473
1-Nitro-5,6-dihydroxy-dihydronaphthalene	5.96	208.0618	[M + H] +	Metabolites	11,954,052	HMDB0060328	C10H9NO4	Benzenoids	2.62	0.0370
Undecanedioic acid	6.11	215.1272	[M − H] −	Xenobiotic exposure	15,816	HMDB0000888	C11H20O4	Lipids and lipid-like molecules	77.07	0.0483
3-Hydroxysebacic acid	5.86	217.1079	[M − H] −	Metabolites	3,017,884	HMDB0000350	C10H18O5	Organic acids and derivatives	2.47	0.0163
Genistein	5.93	271.0610	[M + H] +	Xenobiotic exposure	5,280,961	HMDB0003217	C15H10O5	Phenylpropanoids and polyketides	3.14	0.0475
Biochanin A	5.10	285.0757	[M + H] +	Xenobiotic exposure	5,280,373		C16H12O5	Phenylpropanoids and polyketides	3.24	0.0380
FA 18:3 + 1O	7.34	295.2266	[M + H] +	Metabolites	5,283,011	HMDB0247609	C18H30O3	Lipids and lipid-like molecules	0.34	0.0483
13-OxoODE	8.32	295.2269	[M + H] +	Metabolites	25,791,066		C18H30O3	Lipids and lipid-like molecules	4.24	0.0055
Sphingosine	9.16	300.2897	[M + H] +	Metabolites	5,280,335	HMDB0000252	C18H37NO2	Organic nitrogen compounds	3.06	0.0016
Eicosapentaenoic acid (EPA)	10.52	303.2306	[M + H] +	Metabolites	446,284	HMDB0001999	C20H30O2	Lipids and lipid-like molecules	0.40	0.0295
(3S)-5-[(1R,2R,8aS)-2-Hydroxy-2,5,5,8a-tetramethyl-3,4,4a,6,7,8-hexahydro-1H-naphthalen-1-yl]-3-methylpentanoic acid	10.17	307.2641	[M − H2O + H] +	Xenobiotic exposure	23,844,029		C20H36O3	Lipids and lipid-like molecules	0.22	0.0175
FA 18:3 + 2O	6.85	309.2068	[M − H] −	Metabolites	5,283,017		C18H30O4	Lipids and lipid-like molecules	0.45	0.0196
9(S)-HPODE	8.96	311.2218	[M − H] −	Metabolites	9,548,877	HMDB0006940	C18H32O4	Lipids and lipid-like molecules	0.47	0.0400
5-[(8aS)-2,5,5,8a-Tetramethyl-3-oxo-4a,6,7,8-tetrahydro-4H-naphthalen-1-yl]-3-methylpentanoic acid	7.55	321.2436	[M + H] +	Xenobiotic exposure	24,066,891		C20H32O3	Lipids and lipid-like molecules	0.45	0.0470
(1R,4aS,5R,8aS)-5-(5-Hydroxy-3-methylpentyl)-1,4a-dimethyl-6-methylidene-3,4,5,7,8,8a-hexahydro-2H-naphthalene-1-carboxylic acid	9.38	323.2581	[M + H] +	Xenobiotic exposure	23,955,908		C20H34O3	Lipids and lipid-like molecules	0.48	0.0001
Arachidonic acid	8.02	327.2318	[M + Na] +	Metabolites	444,899	HMDB0001043	C20H32O2	Lipids and lipid-like molecules	0.42	0.0343
Docosahexaenoic acid (DHA)	9.71	329.2484	[M + H] +	Metabolites	445,580	HMDB0002183	C22H32O2	Lipids and lipid-like molecules	0.50	0.0211
8-Hydroxy-9,10-epoxystearic acid	10.53	337.2360	[M + Na] +	Metabolites	53,851,542		C18H34O4	Lipids and lipid-like molecules	2.45	0.0000
FA 18:2 + 4O	10.40	343.2114	[M − H] −	Metabolites	126,457,312		C18H32O6	Lipids and lipid-like molecules	2.33	0.0044
16,17-Dihydroxykauran-18-oic acid	7.84	354.2650	[M + NH4] +	Xenobiotic exposure	5,088,391		C20H32O4	Lipids and lipid-like molecules	0.47	0.0270
Cortisone	8.79	361.1982	[M + H] +	Metabolites	222,786	HMDB0002802	C21H28O5	Lipids and lipid-like molecules	3.30	0.0295
Arachidonyl carnitine	10.98	448.3404	[M + H] +	Metabolites	136,212,424	HMDB0006455	C27H45NO4	Lipids and lipid-like molecule	3.39	0.0087
LysoPC(17:0)	10.31	510.3555	[M + H] +	Metabolites	24,779,463	HMDB0012108	C25H52NO7P	Lipids and lipid-like molecule	2.94	0.0226

*FC* fold change, Aβ_1-42_-induced rats/sham rats

*P*-value of *t* test that calculates the MS features between Aβ_1-42_-induced rats and sham rats

**Table 2:** The results of metabolic pathway analysis

Pathway name	Total^a^	Hits^b^	Raw p^c^	-Log(p)	Impact^d^	Detected metabolites
Phenylalanine metabolism	12	3	0.0013	2.899	0.619	l-Phenylalanine; phenylpyruvate; ortho-hydroxyphenylacetic acid
Phenylalanine, tyrosine, and tryptophan biosynthesis	4	2	0.0023	2.646	0.500	Phenylpyruvate; l-phenylalanine
Arachidonic acid metabolism	36	1	0.0282	1.550	0.333	Arachidonate
Sphingolipid metabolism	21	1	0.0005	3.266	0.045	Sphingosine
Glycerophospholipid metabolism	36	1	0.0147	1.834	0.017	LysoPC(17:0)
Steroid hormone biosynthesis	77	1	0.0266	1.575	0.007	Cortisone
Biosynthesis of unsaturated fatty acids	36	3	0.0054	2.268	0.000	Arachidonate; DHA; EPA
Aminoacyl-tRNA biosynthesis	48	1	0.0196	1.707	0.000	l-Phenylalanine
Metabolism of xenobiotics by cytochrome P450	64	1	0.0287	1.543	0.000	1-Nitro-5,6-dihydroxy-dihydronaphthalene

^a^Total is the total number of compounds in the metabolic pathway

^b^Hits is the number of actual matches in the user uploaded data

^c^Raw *p* is the raw *P* value calculated from the enrichment analysis

^d^Impact is the pathway impact value calculated by the pathway topology analysis

Thirty-three metabolites were then classified in Fig. 5 A and Table 1 using the online software ClassFire (https://cfb.fiehnlab.ucdavis.edu/). Nineteen metabolites belong to lipids and lipid-like molecules, 5 belong to phenylpropanoids and polyketides, 4 belong to benzenoids, 2 belong to organic acids and derivatives, 2 belong to organoheterocyclic compounds, and 1 belong to organic nitrogen compounds. It indicates that most of the significantly different metabolites in hair belong to lipids and lipid-like molecules (Fig. 5A). The perturbed pathways in Aβ_1-42_-induced rats were analyzed using the online software MetaboAnalyst (www.metaboanalyst.ca) (Fig. 5B and Table 2). The cutoff value of 0.1 for pathway impacts score to filter less important pathways [26]. Phenylalanine metabolism, phenylalanine, tyrosine and tryptophan biosynthesis, and arachidonic acid (ARA) metabolism were the significantly perturbed pathways in which the impact scores were > 0.3 (Table 2). For the six metabolites with high correlation with histological/behavioral tests, only l-phenylalanine belongs to phenylalanine metabolism and phenylalanine, tyrosine, and tryptophan biosynthesis is involved in these three perturbed pathways. 8-Hydroxy-9,10-epoxystearic acid’s structure is similar to the metabolites of linoleic acid (LA) metabolism, and 13-oxoODE and FA 18:2 + 4O belong to LA metabolism, lysoPC(17:0) belongs to glycerophospholipid metabolism, and sphingosine belongs sphingolipid metabolism in KEGG pathway (Supplemental Table 2).

### Identification of Metabolites in Aβ_1-42_-Induced Rat-Related Pathways Using HRMS-Based Targeted Metabolomics

To demonstrate whether these three highest impacts perturbed pathways (phenylalanine metabolism, phenylalanine, tyrosine and tryptophan biosynthesis, and ARA metabolism) were possibly Aβ_1-42_-induced, we used the UHPLC-HRMS-based targeted metabolomic analysis to identify the three pathway associated metabolites. Seven metabolites were identified by the targeted-metabolic analysis, in which five metabolites (leukotriene B4 (LTB4), 14,15-DiHETrE (14,15-DHET), 5(S)-HETE, 5(S)-HPETE, and prostaglandin B2 (PGB2)) belonged to ARA metabolism (Table 3). In addition, dehydroepiandrosterone (DHEA) belonged to steroid hormone biosynthesis and dihomo-γ-linolenic acid (DGLA) belonged to LA metabolism which were also identified by the targeted-metabolomic analysis, in which LA was included in unsaturated fatty acid. The correlation between these seven metabolites and histological/behavioral tests are significantly correlation (|*R*|> 0.5) with Thio-S staining, FJB staining, TUNEL staining, HE staining, and Y maze, respectively (Fig. 5C–J). Overall, phenylalanine metabolism (including phenylalanine, tyrosine, and tryptophan biosynthesis), ARA metabolism, and biosynthesis of unsaturated fatty acids (including docosahexaenoic acid (DHA) and eicosapentaenoic acid (EPA) metabolites and the metabolites of LA metabolism) might be the possible pathways of Aβ_1-42_-induced.

**Table 3:** Metabolites in three major perturbed pathways identified by LC-HRMS-based targeted and untargeted metabolomics

Metabolite	RT	m/z	Mode	Pathway	Targeted/untargeted	HMDB	PubChem	Formula	FC (Aβ/Sham)	*P* value
Leukotriene B4 (LTB4)	10.28	337.2373	[M + H] +	Arachidonic acid metabolism; biosynthesis of unsaturated fatty acids	Targeted	HMDB0001085	5,283,128	C20H32O4	2.53	0.0000
Arachidonyl carnitine	7.96	448.3424	[M + H] +	Arachidonic acid metabolism; biosynthesis of unsaturated fatty acids	Untargeted	HMDB0006455	53,477,832	C27H45NO4	3.39	0.0087
14,15-DiHETrE (14,15-DHET)	8.47	337.2383	[M − H] −	Arachidonic acid metabolism; biosynthesis of unsaturated fatty acids	Targeted	HMDB0002265	5,283,147	C20H34O4	0.46	0.0199
Arachidonic acid	8.02	327.2318	[M + Na] +	Arachidonic acid metabolism; biosynthesis of unsaturated fatty acids	Untargeted	HMDB0001043	444,899	C20H32O2	0.42	0.0343
5(S)-HETE	7.85	321.2425	[M + H] +	Arachidonic acid metabolism; biosynthesis of unsaturated fatty acids	Targeted	HMDB0011134	5,280,733	C20H32O3	0.45	0.0470
5(S)-HPETE	11.24	337.2350	[M + H] +	Arachidonic acid metabolism; biosynthesis of unsaturated fatty acids	Targeted	HMDB0004244	5,280,893	C20H32O4	3.04	0.0464
Prostaglandin B2	7.16	333.2067	[M − H] −	Arachidonic acid metabolism; biosynthesis of unsaturated fatty acids	Targeted	HMDB0004236	5,280,881	C20H30O4	0.44	0.0054
Ortho-Hydroxyphenylacetic acid	5.34	151.0390	[M − H] −	Phenylalanine metabolism	Untargeted	HMDB0000669	11,970	C8H8O3	2.45	0.0021
L-Phenylalanine	4.17	166.0863	[M + H] +	Phenylalanine metabolism; phenylalanine, tyrosine and tryptophan biosynthesis	Untargeted	HMDB0000159	6140	C9H11NO2	2.17	0.0224
Phenylpyruvic acid	6.15	163.0391	[M − H] −	Phenylalanine metabolism; phenylalanine, tyrosine, and tryptophan biosynthesis	Untargeted	HMDB0000205	997	C9H8O3	2.54	0.0273
Phenyllactic acid	6.39	165.1274	[M − H] −	Phenylalanine metabolism	Untargeted	HMDB0000779	3848	C9H10O3	3.30	0.0089
Docosahexaenoic acid (DHA)	9.30	329.2473	[M + H] +	DHA metabolites; biosynthesis of unsaturated fatty acids	Untargeted	HMDB0002183	445,580	C22H32O2	0.50	0.0211
Eicosapentaenoic acid (EPA)	10.56	303.2318	[M + H] +	EPA metabolites; biosynthesis of unsaturated fatty acids	Untargeted	HMDB0001999	446,284	C20H30O2	0.40	0.0295
8-Hydroxy-9,10-epoxystearic Acid	10.53	337.2360	[M + Na] +	Linoleic acid metabolism Biosynthesis of unsaturated fatty acids	Untargeted		53,851,542	C18H34O4	2.45	0.0000
13-OxoODE	8.32	295.2269	[M + H] +	Linoleic acid metabolism Biosynthesis of unsaturated fatty acids	Untargeted		25,791,066	C18H30O3	4.24	0.0055
9(S)-HPODE	8.96	311.2218	[M − H] −	Linoleic acid metabolism Biosynthesis of unsaturated fatty acids	Untargeted	HMDB0006940	9,548,877	C18H32O4	0.47	0.0400
Dihomo-γ-linolenic acid (DGLA)	9.367	307.26318	[M + H] +	Linoleic acid metabolism Biosynthesis of unsaturated fatty acids	Targeted	HMDB0002925	5,280,581	C20H34O2	0.22	0.0175
FA 18:2 + 4O	10.40	343.2114	[M − H] −	Linoleic acid metabolism Biosynthesis of unsaturated fatty acids	Untargeted		126,457,312	C18H32O6	2.33	0.0044
FA 18:3 + 1O	7.34	295.2266	[M + H] +	Biosynthesis of unsaturated fatty acids	Untargeted	HMDB0247609	5,283,011	C18H30O3	0.34	0.0483
FA 18:3 + 2O	6.85	309.2068	[M − H] −	Biosynthesis of unsaturated fatty acids	Untargeted		5,283,017	C18H30O4	0.45	0.0196
Cortisone	8.79	361.1982	[M + H] +	Steroid hormone biosynthesis	Untargeted	HMDB0002802	222,786	C21H28O5	3.30	0.0295
Dehydroepiandrosterone (DHEA)	8.262	289.21603	[M + H] +	Steroid hormone biosynthesis	Targeted	HMDB0000077	5881	C19H28O2	2.79	0.0178

## Discussion

In this study, we performed an untargeted metabolomic analysis to discover the perturbation of differential metabolites, pathways, and potential AD biomarkers using hair samples from nontransgenic AD rats and sham rats. To investigate the potential pathway of Aβ_1-42_ effects, the metabolites in the perturbed pathway were validated using a targeted metabolomic analysis. Hair is in the integumentary system and is easy to collect. Because endogenous metabolites in the blood are incorporated into hair through the hair follicle and are distributed in the hair as the hair grows, hair metabolomics can be applied for long-term and stable biological monitoring [14–16]. It has been reported that hair can serve as a long-term storage matrix for toxicant metabolites [27, 28], and valuable information regarding exposure time can be obtained from segmental hair analysis [15]. In this study, we collected hair from Aβ_1-42_-induced and sham rats, detected hair metabolites using UHPLC-HRMS, and identified the differential metabolites between the Aβ_1-42_-induced and sham rats (Fig. 1).

Previous studies have reported behavioral effects of injection of Aβ into the brain, showing deficits in the acquisition of reference memory tasks, consolidation of learning, and spatial and working memory [29–31]. In the present study, the behavioral test and histological assay demonstrated that *i.c.v.* Aβ_1-42_ injection had significant effects on developing neuropathological signs of AD as well as spatial learning memory and motor function (Figs. 2 and 3). The significant changes of hair MS peaks in Aβ_1-42_-induced rats compared with sham rats were filtered by volcano plots (Fig. 4A and B). Peaks with a |fold change|≥ 2 and *p* ≤ 0.05 were subjected to MS/MS. Thirty-three chemicals were identified (Table 1). Six metabolites showed a high correlation (|*R*|> 0.7) with the histological and behavioral test, including l-phenylalanine, 8-hydroxy-9,10-epoxystearic acid, lysoPC(17:0), sphingosine, 13-OxoODE, and FA 18:2 + 4O (Fig. 4C–J). Nineteen of thirty-three metabolites were lipid and lipid-like molecules indicating that non-polar molecules were the main compounds detected in hair samples (Fig. 5A). Phenylalanine metabolism, ARA metabolism, and biosynthesis of unsaturated fatty acid were high impacts in Aβ_1-42_-induced rats based on pathway analysis in the online software MetaboAnalyst 5.0 (Fig. 5B) and targeted-metabolomic analysis (Table 2 and Supplemental Table 2). Seven metabolites detected by targeted analysis show significant correlation (|*R*|> 0.5) with histological/behavioral tests, including ARA metabolites (LTB4, 14,15-DHET, 5(S)-HETE, 5(S)-HPETE, and PGB2), metabolites of steroid hormone biosynthesis (DHEA), and LA metabolism (DGLA) (Fig. 5C–J). Forty metabolites are identified by untargeted- and targeted-metabolomic analysis, in which twenty are included in these three perturbed pathways. Four metabolites belong to phenylalanine metabolism, seven belong to ARA metabolism, and nine belong to the biosynthesis of unsaturated fatty acid, including EPA, DHA, five of LA metabolism, and two of unsaturated fatty acid (Table 3). In addition, two metabolites belong to steroid hormone biosynthesis (Table 3). Overall, twenty metabolites are involved in these three pathways.

### Phenylalanine Metabolism

The levels of l-phenylalanine, phenylpyruvic acid, phenyllactic acid, and ortho-hydroxyphenylacetic acid, which belong to the phenylalanine metabolism pathway, in the hair were measured and found to be significantly different between Aβ_1-42_-induced rats and sham rats. Phenylalanine, an essential amino acid for the human body, is converted to tyrosine by phenylalanine hydroxylase, and tyrosine produces dopamine, a neurotransmitter. A large amount of phenylalanine accumulated in the body results in producing many toxic metabolites [32]. It is reported that phenylalanine metabolism is dysregulated in the human hippocampus and related to AD’s pathological changes [33]. In addition, some studies have found that patients with phenylketonuria (PKU), a rare disease with cranial nerve damage caused by abnormal phenylacetone metabolism, contain higher amounts of Aβ_1-42_ and tau in the CSF [34]. Horster and colleagues show that phenylalanine reduces synaptic density in mixed cortical cultures from mice [35], as well as Preissler and colleagues report that phenylalanine induces oxidative stress and reduces the viability of rat astrocytes [36]. It is reported that a single amino acid of phenylalanine can self-assemble into fibrils with amyloid-like morphology, which can be recognized and be neutralized the cytotoxicity by antibodies, and be presented in the hippocampus of model mice and in the parietal cortex brain tissue of PKU patients [37].

In the present study, these four metabolites were upregulated in the hair samples of Aβ_1-42_-induced rats compared with sham rats (Table 3). Studies have shown that a lower concentration of phenylalanine is detected in the serum of AD patients [13], but Wissmann and colleagues report that high serum phenylalanine is associated with AD [38]. In addition, Badhwar and colleagues report phenylalanine is higher in the CSF of AD patients [39]; Liu and colleagues report that phenylalanine and phenylpyruvic acid are upregulated in the hippocampus of AD patients [40], indicating that the expression level of phenylalanine is fluctuating in blood sample, and hair can stably reflect the expression level of phenylalanine in brain tissue of CSF.

### Arachidonic Acid Metabolism

ARA belongs to non-essential fatty acid, is a polyunsaturated ω-6 fatty acid, is present in the phospholipids of cell membranes, and is abundant in the brain, muscles, and liver. In the present study, LTB4, arachidonyl carnitine, 14,15-DHET, ARA, 5(S)-HETE, 5(S)-HPETE, and PGB2 were detected by targeted and untargeted metabolomic analysis (Table 3). Except LTB4, arachidonyl carnitine, and 5(S)-HPETE, the other four metabolites are downregulated in Aβ_1-42_-induced rats compared with sham rats. ARA convers to EETs, while EETs convert to inactive DHET by soluble epoxide hydrolase (sEH), in which 14,15-DHET is down-regulated in the plasma of AD patients in previous study [41]. In addition, ARA is upregulation in the plasma of AD patients but downregulated in the CSF of AD patients [41]. PGB2 is a prostanoid and is reported to induce vasoconstriction on vascular smooth muscle tone [42]. It was reported that prostaglandins exhibit neurotoxic or neuroprotective effects by acting on specific G protein–coupled receptors, suggesting that prostaglandins in AD may help to elucidate their neuroprotective effects [43]; however, there are no reports related to PGB2 and AD, showing the correlation between PGB2 and AD was unclear. LTB4, a pro-inflammatory mediator [44], has been reported to increase Aβ production in vitro, whereas treatment with an ARA pathway inhibitor converts dihomo-γ-linolenic/linoleic acid to ARA and resulted in inhibiting Aβ production [45]. In the present study, 5(S)-HPETE, the upstream of LTB4, is both upregulated with LTB4 in Aβ_1-42_-induced rats; in addition, 5(S)-HETE, another downstream of 5(S)-HPETE, is downregulated. It indicates that the downregulation of ARA and the upregulation of 5(S)-HPETE and LTB4 in the hair of Aβ_1-42_-induced rats reflect Aβ_1-42_ induced ARA production of LTB4, not of 14,15-DHET, 5(S)-HETE, and PGB2.

Arachidonoyl carnitine, which belongs to acylcarnitines, is upregulated in the hair of Aβ_1-42_-induced rats and plays a role in ARA metabolism. Long-chain fatty acids are catalyzed to long-chain fatty acyl-CoA and then converted to acylcarnitines, the first step in the transport of long-chain fatty acids from the cytoplasm to the mitochondrial matrix where oxidation occurs [46]. It has been reported that long-chain acylcarnitines are increased in the plasma samples of AD patients [13].

### Biosynthesis of Unsaturated Fatty Acid

Unsaturated fatty acids, including monounsaturated (MUFA) and polyunsaturated fatty acids (PUFA), have one or more double bonds. It is known that only two types of fatty acids are essential to humans and can only be obtained from food: α-linolenic acid (ALA, a kind of ω-3 fatty acid) and LA (a kind of ω-6 fatty acid). ω-3 and ω-6 fatty acids belong to unsaturated fatty acids. There are three ω-3 fatty acids involved in human physiology, including ALA, EPA, and DHA, of which ALA cannot be synthesized in mammals and can only be obtained from food, but EPA and DHA can be formed by ALA or be obtained from food. LA belongs to ω-6 fatty acid and is unable to be synthesized in mammals and can only be obtained from food. In addition, ARA also belongs to ω-6 fatty acid. In the present study, 9 metabolites belong to the biosynthesis of unsaturated fatty acids, including EPA and DHA, LA metabolism (8-hydroxy-9,10-epoxystearic acid, 13-OxoODE, 9(S)-HPODE, and DGLA), FA 18:3 + 1O, FA 18:2 + 4O, and FA 18:3 + 2O, whereas only 8-hydroxy-9,10-epoxystearic acid, 13-OxoODE, and FA 18:2 + 4O are upregulation in Aβ_1-42_-induced rats.

It is reported that 13-OxoODE, one of the oxidized LA metabolites, is elevated in blood of AD patients [47]. 8-Hydroxy-9,10-epoxystearic acid is a metabolite of C18 fatty acid converted by linoleate diol synthase [48], and its structure is similar with the metabolites of LA metabolism. Oemer and colleagues reported that the brain displays a unique and diverse signature of long FA-rich acyl chains (FA 20:4 and FA 22:6), unlike other mammalian tissues that display a more uniform acyl chain pattern, defined by preferential incorporation of LA (18:2) [49]. However, PUFA oxidation contributes to the progressive neuronal loss associated with neurodegenerative diseases, such as AD and PD [49]. Hence, the upregulation of FA18:2 + 4O, an oxidized LA in Aβ_1-42_-induced rats’ hair, may be the possible biomarker that reflected the PUFA oxidation in the brain of AD rats. FA 18:3 + 1O and FA 18:3 + 2O belong to the oxidation of ALA, whereas ALA is an isomer of γ-linolenic acid (GLA) and is anti-inflammation. Unlike ALA (ω-3 fatty acid), GLA belongs to ω-6 fatty acid. It is reported that ALA inhibits Tau aggregation and regulates Tau conformation [50]. 9(S)-HPODE is also the metabolite of LA metabolism and was downregulated in the present study and previous study [41]. DGLA is also the metabolite of LA metabolism and cannot yield leukotrienes, but it can inhibit the formation of pro-inflammatory leukotrienes from ARA to anti-inflammatory [51]. It is demonstrated that DHA and EPA exhibit neuroprotective properties that improve or treat neurodegenerative and neurological disorders [52]. Especially, DHA is quantitatively the most important ω-3 PUFA in the brain [52]. It is demonstrated that DHA and EPA are downregulated in the CSF of AD patients [39]. In the present study, DHA and EPA are decreased in the hair of Aβ_1-42_-induced rats.

### Novel and Potential AD Biomarker Candidates

On the other hand, four metabolites identified by untargeted metabolomic analysis, sphingosine, lysoPC(17:0), cortisone, and dehydroepiandrosterone are also associated with AD, as well as sebacic acid and 3-hydroxysebacic acid are related to brain injury (Table 1). Herein, we discussed the implications of these metabolites on AD and other neurodegenerative diseases.

Neurodegenerative diseases, such as AD, Parkinson’s disease (PD), Huntington’s disease (HD), and amyotrophic lateral sclerosis (ALS), are characterized by a complex combination of pathological features such as axonal damage, demyelination, gliosis, neuroinflammation, synaptic toxicity, and neuronal death, which result in neurobehavioral deficits [53]. The toxic protein aggregation served as the hallmark of pathology in AD (e.g., Ab and tau), PD (e.g., a-synuclein), and ALS (e.g., TAR DNA binding protein 43, TDP-43).

The homeostasis of membrane sphingolipids is essential to maintain the neuron and myeline sheath function and structural integrity. The sphingolipids involved in neurodegeneration are sphingosines (e.g., sphinganine, sphingosine, sphingosine-1-phosphate), ceramide, derived molecules (e.g., galactosylceramide, glucosylceramide, and sphingomyelin), and gangliosides [53]. Sphingolipids are the primary target of Ab and contribute to Ab conformation changes, which are directly involved in Ab-induced neurotoxicity and implicated in brain aging and AD [54]. Sphingosine, which belongs to sphingolipid metabolism, is upregulated in the hair of Aβ_1-42_-induced rats, whereas phytosphingosine, dihydrosphingosine, and hexadecasphinganine, the other metabolites of sphingolipid metabolism, are downregulated in the blood of AD patients [55]. Sphingolipids also participate in the stability of α-synuclein. Recent studies have demonstrated that sphingolipid metabolism impairment in PD is linked to protein clearance system (e.g., autophagy) dysfunction. After that, the α-synuclein accumulation in neurons and these aggregates present in the inclusion of Lewy bodies which are associated with PD [56]. Several studies have also revealed significant changes in sphingolipid pathway lipids and related enzymes in the PD brain that are also presented in CSF and blood [57]. Therefore, the sphingolipid metabolism alterations of PD may be served as a potential diagnostic feature. HD is a dominantly inherited neurodegenerative disorder. Lipid metabolism disturbance was also found in HD. Di Pardo et al. demonstrated a significantly upregulated expression of Sphingosine1-phosphate Lyase1 and downregulated sphingosine kinase 1 in the post-mortem brains of patients with HD [58]. The upregulated Sphingosine1-phosphate Lyase1 may reduce the availability of the bioactive lipid and enhance the release of hexadecenal resulting in induced cytotoxicity [58, 59]. ALS is characterized by motor neuron degeneration in the spinal cord, motor cortex, and brain stem. Several studies revealed that patients with ALS have a high level of sphingolipids (glucosylceramide, ceramide, galactosylceramide, sphingomyelin, etc.) in the lumbar spinal cord [59, 60], indicating that altered metabolism of sphingolipids could be involved in ALS pathogenesis.

Lysophosphatidylcholine (LysoPC) is a lipid biomolecule derived by the cleaving of phosphatidylcholine (PC) via phospholipase A2, which is essential to membrane permeability [61]. LysoPC could transport long-chain fatty acids such as DHA across the BBB into the brain [62]. In contrast, LysoPC could also activate the inflammasome and subsequently induce pyroptosis exaggerating the neurodegeneration [63]. LysoPC(17:0) (lysophosphatidylcholine 17:0) is upregulated in the hair of Aβ_1-42_-induced rats and belongs to glycerophospholipid metabolism, is formed from phosphatidylcholine, and also can convert back to phosphatidylcholine. Because phosphatidylcholine is upstream of ARA in ARA metabolism, the upregulation of lysoPC(17:0) may associate with ARA metabolism. It is reported that several lysoPCs are upregulated in the plasma of AD patients [64]. In PD, Farmer et al. have demonstrated the LysoPC upregulation within the substantia nigra in a specific neurotoxin 6-hydroxydopamine (6-OHDA)–induced PD rat model [65]. The dysregulation of LysoPC may be involved in a-synuclein-mediated neuroinflammation in the pathogenesis of PD. The role of LysoPC in the pathogenesis of HD is still unclear. Comprehensive lipidomics analysis of blood serum from ALS patients established a significant increase in PC, LysoPC, and sphingomyelin metabolism in ALS compared with controls [66, 67].

Cortisone and DHEA, two metabolites that belong to steroid hormone biosynthesis, are upregulated in Aβ_1-42_-induced rats identified by targeted- and untargeted-metabolomic analysis. Cortisone, converted from cortisol, is reported that cortisol is significantly increased in the serum of AD patients to be the independent biomarkers for AD progression [68]. In addition, cortisol is upregulated in the CSF of AD patients [39]. DHEA, an endogenous steroid hormone synthesized by 17α-hydroxypregnenolone, can act as a prohormone for progestogens, mineralocorticoids, glucocorticoids, androgens, and estrogens. DHEA is also called neurosteroid because it can regulate neuronal excitability and function through the N-methyl-d-aspartate receptor activation, a fundamental mechanism of cognition function [69]. It is reported that DHEA was significantly lower in AD patients compared to controls, and the DHEA levels were significantly correlated to dehydroepiandrosterone sulfate (DHEAS, a metabolite of DHEA) levels in both control and AD conditions [70]. In addition, DHEAS enhances the blood–brain barrier (BBB) and tight junction protein expression in the endothelial cells of brain [71]. A few studies examined alterations in DHEA and DHEAS levels in PD. While DHEAS was lower in AD, no changes were observed in DHEAS plasma levels in PD patients when compared with healthy controls [72]. Elevated cortisol levels and downregulated DHEA levels in the patient’s plasma were observed in HD [73]. In animal studies, prolonged exposure to high serum cortisol leads to irreversible hippocampal dysfunction [73]. In ALS, patients exhibited higher levels of a higher DHEAS/cortisol ratio and worse prognoses [74]. The alterations of cortisol and DHEA may subsequently affect the neuronal activity in the brain, but the underlying mechanisms remain unclear. Long-term studies are necessary to assess the correlation between neurosteroids and neurodegenerative diseases.

3-Hydroxysebacic acid is an α, ω-dicarboxylic acid, which is sebacic acid with a hydroxyl substituent at the 3-position. Sebacic acid and 3-hydroxysebacic acid are upregulated in Aβ_1-42_-induced rats. Sebacic acid (sebacate, decanedioic acid), a saturated, straight-chain naturally occurring dicarboxylic acid, is a normal urinary acid. It is reported that sebacic acid is increased in the urine of multiple acyl-CoA-dehydrogenase deficiency patients, which is a disease of metabolic disorders due to deficiency of either electron transfer flavoprotein or electron transfer flavoprotein ubiquinone oxidoreductase [75]. High levels of 3-hydroxysebacic acid have been detected in the urine of patients with ketoacidosis. Large amounts of ketone bodies can be toxic to the brain, such as ketoacidosis, a dangerous complication that can occur in diabetes or alcoholism [76].

**Fig. 6:**
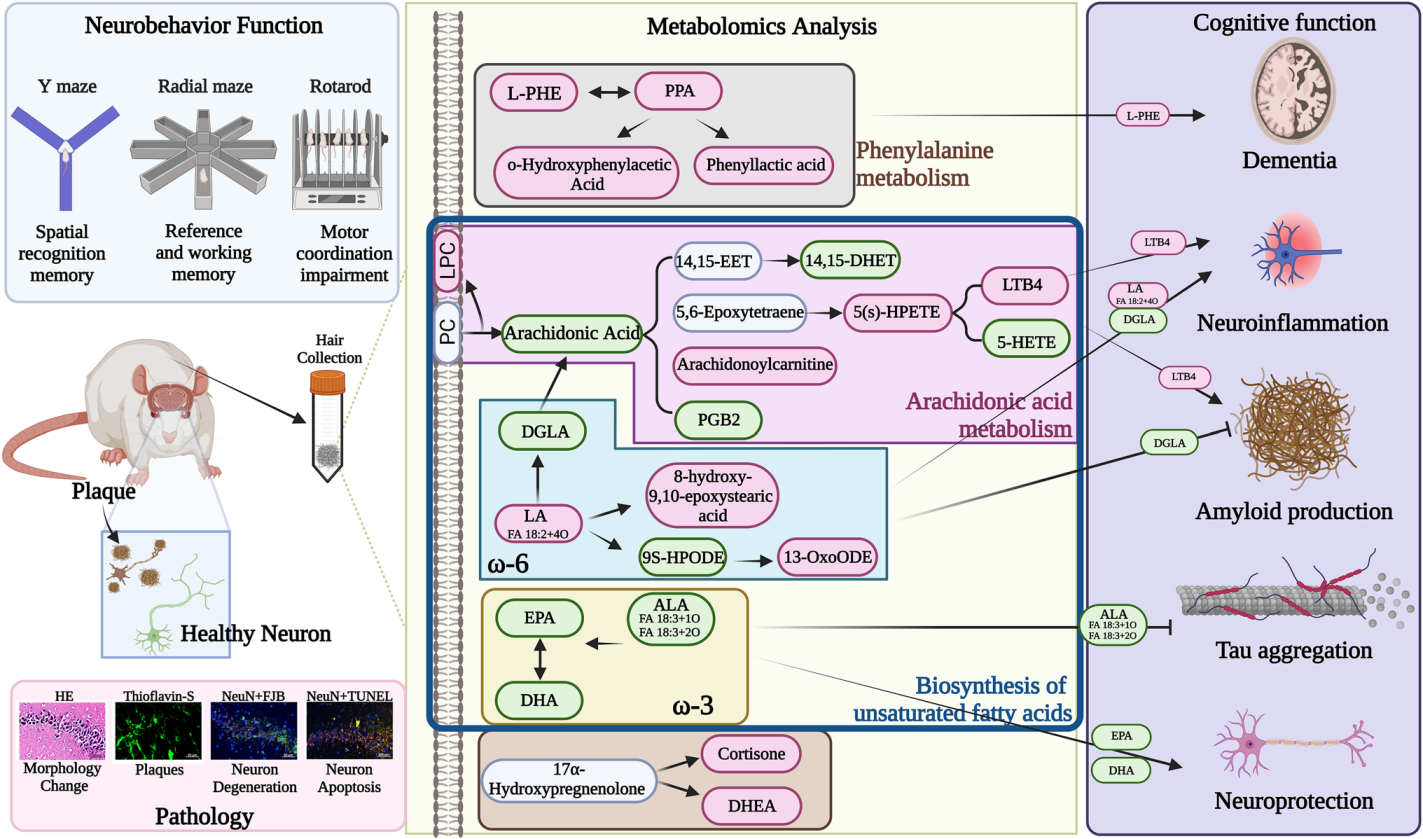
Overview of the altered metabolites and perturbed pathways in Aβ_1-42_-induced rats. The changes affected by Aβ_1-42_ are indicated by the pink and green boxes. Pink box: upregulation in Aβ_1-42_-induced rats compared with sham rats; green box: downregulation in Aβ_1-42_-induced rats compared with sham rats; blue box: not analyzed in UHPLE-HRMS analysis. (ALA, α-linolenic acid; DGLA, dihomo-γ-linolenic acid; L-PHE, l-phenylalanine; PC, phosphatidylcholine; PEA, phenethylamine; PGB2, prostaglandin B2; PPA, phenylpyruvic acid; LPC: lysophosphatidylcholine; LTB4, leukotriene B4; LA, linoleic acid)

In summary (Fig. 6), we employed untargeted metabolomics to identify potential metabolic biomarkers and targeted metabolomic analysis to validate the perturbed metabolites in the hair of AD rats. Following univariate analysis, 33 metabolites were found to be differentially altered in the hair of AD rats. Pathway analysis showed that phenylalanine metabolism, arachidonic acid metabolism, and biosynthesis of unsaturated fatty acid metabolism were the most relevant pathways associated with AD. Our study showed significant associations between these metabolite levels and amyloid plaque, neuronal apoptosis, neurodegeneration, and cognitive performance. As mentioned above, previous studies have shown that MUFA and PUFA metabolism was perturbed in the brain of AD patients. Our results showed a reduction in Omega-3 PUFA biosynthesis, including ALA, DHA, and EPA, in AD rats, which means the FA metabolism deficits and supplementation with DHA or EPA may be a benefit to improve cognitive function.

The Chicago Health and Aging Project reported that a high dietary intake of omega-6 PUFA (including LA and AA) could reduce the risk for AD [77]. Our omega-6 PUFA data showed DGLA and AA reduction and LA elevation in AD rats’ hair. In contrast, previous studies showed no association between DGLA or AA and incident AD or cognitive impairment [78]. However, the relationship between DGLA, AA, and AD study is still limited and needs further investigation. LA is BBB penetrable, but most of the LA is not converted to AA in the brain. LA is broken down by fatty acid β-oxidation to generate energy but accompanies by ROS elevation, which could be decreased bioenergetic function in AD [79]. Therefore, LA could serve as an ideal target for further investigation of AD-related bioenergetic decline. AA is an agonist of inflammatory pathways which may be involved in the neuroinflammation of AD pathogenesis. However, the potential effects of dietary AA and various AA metabolites on amyloidosis in the AD brain are still unclear. The LTB4, one of the AA metabolites that act pro-inflammatory function, is upregulated in the hair of AD rats in the present study. This is also consistent with the data in patients in which higher expression of LTB4 in CSF of both AD and mild cognitive impairment patients and positively correlated to the levels of Ab 42 in AD patients [80]. Based on the results of the current study, we propose that inhibitors for LTB4 syntheses may be therapeutically beneficial for the intervention of AD.

Finally, our untargeted metabolomics shows that the phenylalanine metabolism in hair is involved in AD pathology. Targeted metabolomics quantified the content of l-phenylalanine upregulated in the hair of AD rats, which is correlated with amyloid plaque formation (evidenced by Thio-S stain). This indicates the aberrant phenylalanine metabolism provides new clues for AD pathogenesis and potential biomarkers or drug targets for AD.

## Conclusion

In this study, we used HRMS-based untargeted and targeted metabolomics to analyze the hair of Aβ_1-42_-induced rats. Thirty-three metabolites are identified by untargeted metabolomic analysis, including 20 metabolites and 13 xenobiotic exposures. Nineteen of thirty-three metabolites are lipid and lipid-like molecules, indicating hair can well reflect the expression of non-polar molecules. In addition, seven metabolites associated with the perturbed pathways are identified by targeted metabolomics. Overall, forty metabolites are identified in the present study. Among them, twenty metabolites are involved in the perturbed pathways (Fig. 6), in which 7 metabolites belong to ARA metabolism, 4 belong to phenylalanine metabolism, and 9 belong to biosynthesis of unsaturated fatty acid (including linoleic acid metabolism, DHA, and EPA), indicating Aβ_1-42_ induces these pathway alternations. Furthermore, 6 metabolites reported in previous studies are detected and significantly altered in the hair of Aβ_1-42_-induced rats, showing moderate (0.4 <|*R*|< 0.7, including ARA, DHA, EPA, and cortisone) to high correlation (|*R*|> 0.7, including l-phenylalanine and sphingosine) with histological/behavioral tests (Supplemental Table 2). Among them, ARA, DHA, EPA, l-phenylalanine, and cortisone show the similar changing trend in the CSF of AD patients in previous studies. This outcome demonstrates that hair can be a biospecimen to detect the alternation of ARA metabolism and AD markers, and these metabolites have the potential to serve as novel AD biomarker candidates. We are hopeful that our novel AD biomarker candidates may be applied in the diagnosis of AD after the development of a quantitative analytical method, evaluation of inter-laboratory reproducibility, larger-scale verification projects, and much more, and eventually benefit the welfare of AD patients.

## Limitation

AD biomarker candidates were discovered in hair samples of an Aβ_1-42_-induced AD rat model; however, the applicability of these candidates in the hair of AD patients requires further validation. In addition, we only analyzed hair samples collected 5 weeks after Aβ_1-42_ injection; thus, the discovered AD candidate markers are early pathological metabolites. On the other hand, the different stages of pathological AD candidate markers can be discovered in the hair samples after different days of Aβ_1-42_ injection. In future studies, AD biomarker candidates for the mid-and late-stages can be identified and validated in hair samples later after injection of Aβ_1-42_. Furthermore, the difference in biomarker candidates among hair, tissues, and biofluids is also valuable data to reveal the potential metabolism pathway of hair AD biomarkers, which is not involved in the current study design. The further implementation work would use hair and compare it with the metabolomics of the brain and biofluids in the near future, which will provide the metabolomic differences as well as similarities between the hair and brain or biofluids, contributing to clinical AD diagnosis and prognosis.

## Supplementary Information

Below is the link to the electronic supplementary material.

Supplementary file1 (DOCX 55 KB)

## Data Availability

The authors confirm that the data supporting the findings of this study are all available within the article.

## Abbreviations

AD: Alzheimer’s disease
ALA: α-Linolenic acid
ARA: Arachidonic acid
Aβ_1-42_: Amyloid β protein fragment 1–42
CSF: Cerebrospinal fluid
DDA: Data-dependent acquisition
DGLA: Dihomo-γ-linolenic acid
DHA: Docosahexaenoic acid
DHEA: Dehydroepiandrosterone
EPA: Eicosapentaenoic acid
GLA: γ-Linolenic acid
HCD: Higher-energy collisional dissociation
HMDB: Human Metabolome Database
LA: Linoleic acid
LTB4: Leukotriene B4
LV: Lateral ventricles
MoNA: MassBank of North America
MUFA: Monounsaturated fatty acid
PET: Positron emission tomography
PGB2: Prostaglandin B2
PUFA: Polyunsaturated fatty acid
TCA: Tricarboxylic acid
UHPLC-HRMS: Ultra-high-performance liquid chromatography-high-resolution mass spectrometry
