# Supplementary material for: Rat Hair Metabolomics Analysis Reveals Perturbations of Unsaturated Fatty Acid Biosynthesis, Phenylalanine, and Arachidonic Acid Metabolism Pathways Are Associated with Amyloid-β-Induced Cognitive Deficits
Source: Mol Neurobiol. 2023 Apr 25;60(8):4373–95. doi: 10.1007/s12035-023-03343-6 (PMC10293421; doi:10.1007/s12035-023-03343-6)
Supplement: Supplementary file 1 — Supplementary file1 (DOCX 55 KB) [file 12035_2023_3343_MOESM1_ESM.docx]

**Supplemental Table 1. Correlations between histological staining and behavioral test.**

| **Histological staining** | **Behavioral Test** | **R^2^** | **R** | **F test(Dfn,Dfd)** | **P value** |
| --- | --- | --- | --- | --- | --- |
| **HE** | **Radial Maze- Time** | 0.2047 | 0.4524 | F(1, 18)=4.632 | 0.0452 |
|  | **Radial Maze- Working Memory** | 0.1699 | 0.4122 | F(1, 18)=3.683 | 0.071 |
|  | **Y Maze** | 0.5572 | -0.7465 | F(1, 18)=22.65 | 0.0002 |
|  | **Rotarod- Time** | 0.2166 | -0.4654 | F(1, 18)=4.976 | 0.0387 |
|  | **Rotarod- Speed** | 0.2251 | -0.4745 | F(1, 18)=5.228 | 0.0346 |
| **Thio-S** | **Radial Maze- Time** | 0.0241 | 0.1551 | F(1, 18)=0.4440 | 0.5137 |
|  | **Radial Maze- Working Memory** | 0.0146 | 0.1206 | F(1, 18)=0.2658 | 0.6125 |
|  | **Y Maze** | 0.3751 | -0.6125 | F(1, 18)=10.80 | 0.0041 |
|  | **Rotarod- Time** | 0.0073 | -0.0857 | F(1, 18)=0.1332 | 0.7194 |
|  | **Rotarod- Speed** | 0.0052 | -0.0720 | F(1, 18)=0.09387 | 0.7628 |
| **TUNEL** | **Radial Maze- Time** | 0.1631 | 0.4039 | F(1, 18)=3.507 | 0.0774 |
|  | **Radial Maze- Working Memory** | 0.0923 | 0.3038 | F(1, 18)=1.831 | 0.1928 |
|  | **Y Maze** | 0.4982 | -0.7058 | F(1, 18)=17.87 | 0.0005 |
|  | **Rotarod- Time** | 0.4428 | -0.6654 | F(1, 18)=14.31 | 0.0014 |
|  | **Rotarod- Speed** | 0.4492 | -0.6702 | F(1, 18)=14.68 | 0.0012 |
| **FJB** | **Radial Maze- Time** | 0.1868 | 0.4322 | F(1, 18)=4.134 | 0.057 |
|  | **Radial Maze- Working Memory** | 0.1550 | 0.3937 | F(1, 18)=3.301 | 0.0859 |
|  | **Y Maze** | 0.5681 | -0.7537 | F(1, 18)=23.67 | 0.0001 |
|  | **Rotarod- Time** | 0.4565 | -0.6757 | F(1, 18)=15.12 | 0.0011 |
|  | **Rotarod- Speed** | 0.4505 | -0.6712 | F(1, 18)=14.76 | 0.0012 |

**Supplemental Table 2. Pathway and correlations of the naturally occurring metabolites**

| Compound Name | Cognitive Function | | | | | Pathology | | | | | Biological Function |
| --- | --- | --- | --- | --- | --- | --- | --- | --- | --- | --- | --- |
|  | Test | R^2^ | R | F test(Dfn,Dfd) | *p* | Test | R^2^ | R | F test(Dfn,Dfd) | *p* |  |
| Metabolites detected by untargeted metabolomics | | | | | | | | | | | |
| Ortho-Hydroxyphenylacetic Acid | Y Maze | 0.3738 | -0.6114 | F(1, 18)=10.75 | 0.004 | HE | 0.2674 | 0.5171 | F(1, 18)=6.569 | 0.02 | Phenylalanine metabolism |
|  |  |  |  |  |  | ThioS | 0.2646 | 0.5144 | F(1, 18)=6.477 | 0.02 |  |
|  |  |  |  |  |  | NeuN+TUNEL | 0.2375 | 0.4873 | F(1, 18)=5.607 | 0.02 |  |
| Phenylpyruvic Acid |  |  |  |  |  | HE | 0.3926 | 0.6266 | F(1, 18)=11.64 | 0.003 | Phenylalanine metabolism |
|  |  |  |  |  |  | NeuN+TUNEL | 0.2968 | 0.5448 | F(1, 18)=7.598 | 0.01 | Phenylalanine, tyrosine and tryptophan biosynthesis |
| L-Phenylalanine | Y Maze | 0.2633 | -0.5131 | F(1, 18)=6.433 | 0.02 | HE | 0.2692 | 0.5188 | F(1, 18)=6.631 | 0.02 | Phenylalanine metabolism |
|  |  |  |  |  |  | ThioS | 0.5851 | 0.7649 | F(1, 18)=25.39 | <0.0001 | Phenylalanine, tyrosine and tryptophan biosynthesis |
|  |  |  |  |  |  | NeuN+FJB | 0.2686 | 0.5183 | F(1, 18)=6.612 | 0.02 | Aminoacyl-tRNA biosynthesis |
| Elcosapentaenoic Acid (EPA) |  |  |  |  |  | ThioS | 0.2121 | -0.4605 | F(1, 18)=4.845 | 0.04 | Biosynthesis of unsaturated fatty acids |
|  |  |  |  |  |  | NeuN+TUNEL | 0.2277 | -0.4772 | F(1, 18)=5.308 | 0.03 |  |
| Arachidonic Acid |  |  |  |  |  | ThioS | 0.1971 | -0.4440 | F(1, 18)=4.418 | 0.05 | Biosynthesis of unsaturated fatty acids |
|  |  |  |  |  |  | NeuN+TUNEL | 0.1971 | -0.4440 | F(1, 18)=4.419 | 0.05 | Arachidonic acid metabolism |
| 1-Nitro-5,6-dihydroxy-dihydronaphthalene | Y Maze | 0.2364 | -0.4862 | F(1, 18)=5.573 | 0.03 |  |  |  |  |  | Metabolism of xenobiotics by cytochrome P450 |
| Sphingosine | Y Maze | 0.3967 | -0.6298 | F(1, 18)=11.84 | 0.003 | HE | 0.2809 | 0.5300 | F(1, 18)=7.031 | 0.02 | Sphingolipid metabolism |
|  |  |  |  |  |  | ThioS | 0.6145 | 0.7839 | F(1, 18)=28.69 | <0.0001 |  |
|  |  |  |  |  |  | NeuN+TUNEL | 0.3263 | 0.5712 | F(1, 18)=8.72 | 0.009 |  |
|  |  |  |  |  |  | NeuN+FJB | 0.4166 | 0.6454 | F(1, 18)=12.85 | 0.002 |  |
| LysoPC(17:0) | Y Maze | 0.3109 | -0.5576 | F(1, 18)=8.121 | 0.01 | ThioS | 0.7559 | 0.8694 | F(1, 18)=55.73 | <0.0001 | Glycerophospholipid metabolism |
|  |  |  |  |  |  | NeuN+FJB | 0.1996 | 0.4468 | F(1, 18)=4.487 | 0.05 |  |
| Docosahexaenoic acid (DHA) | Y Maze | 0.2116 | 0.4600 | F(1, 18)=4.832 | 0.0413 | NeuN+TUNEL | 0.1982 | -0.4452 | F(1, 18)=4.449 | 0.0492 | Biosynthesis of unsaturated fatty acids |
|  |  |  |  |  |  | NeuN+FJB | 0.2992 | -0.5470 | F(1, 18)=7.684 | 0.0126 |  |
| Cortisone | Y Maze | 0.3704 | -0.6086 | F(1, 18)=10.59 | 0.004 | ThioS | 0.4131 | 0.6427 | F(1, 18)=12.67 | 0.002 | Steroid hormone biosynthesis |
|  |  |  |  |  |  | NeuN+FJB | 0.2776 | 0.5269 | F(1, 18)=6.917 | 0.02 |  |
| Arachidonyl Carnitine | Y Maze | 0.3383 | -0.5816 | F(1, 18)=9.204 | 0.007 | NeuN+TUNEL | 0.2697 | 0.5193 | F(1, 18)=6.649 | 0.02 | Arachidonic acid metabolism |
|  |  |  |  |  |  | NeuN+FJB | 0.2099 | 0.4581 | F(1, 18)=4.783 | 0.04 |  |
| 8-Hydroxy-9,10-epoxystearic Acid | Y Maze | 0.557 | -0.7463 | F(1, 18)=22.64 | 0.0002 | HE | 0.4415 | 0.6645 | F(1, 18)=10.81 | 0.001 | Linoleic acid metabolism |
|  | Radial Maze-Latency | 0.228 | 0.4775 | F(1, 18)=5.316 | 0.03 | ThioS | 0.4147 | 0.6440 | F(1, 18)=11.99 | 0.002 | Biosynthesis of unsaturated fatty acids |
|  |  |  |  |  |  | NeuN+TUNEL | 0.6192 | 0.7869 | F(1, 18)=11.54 | <0.0001 |  |
|  |  |  |  |  |  | NeuN+FJB | 0.6543 | 0.8089 | F(1, 18)=6.467 | <0.0001 |  |
| 13-OxoODE | Y Maze | 0.3934 | -0.6272 | F(1, 18)=11.67 | 0.003 | ThioS | 0.5053 | 0.7108 | F(1, 18)=18.38 | 0.0004 | Linoleic acid metabolism |
|  |  |  |  |  |  | NeuN+FJB | 0.333 | 0.5771 | F(1, 18)=8.987 | 0.008 |  |
| 9(S)-HPODE |  |  |  |  |  |  |  |  |  |  | Linoleic acid metabolism |
| FA 18:3+1O |  |  |  |  |  | HE | 0.2336 | -0.4833 | F(1, 18)=5.487 | 0.03 | Biosynthesis of unsaturated fatty acids; α-linolenic acid |
| FA 18:2+4O | Y Maze | 0.5187 | -0.7202 | F(1, 18)=19.4 | 0.0003 | ThioS | 0.4081 | 0.6388 | F(1, 18)=12.41 | 0.002 | Biosynthesis of unsaturated fatty acids; Linoleic acid |
| FA 18:3+2O |  |  |  |  |  | ThioS | 0.2561 | -0.5061 | F(1, 18)=6.197 | 0.02 | Biosynthesis of unsaturated fatty acids; α-linolenic acid |
| Phenyllactic Acid | Radial Maze-Working Memory | 0.2069 | 0.4549 | F(1, 18)=4.696 | 0.04 | ThioS | 0.2577 | 0.5076 | F(1, 18)=6.248 | 0.02 | Phenylalanine metabolism |
|  |  |  |  |  |  | NeuN+FJB | 0.2745 | 0.5239 | F(1, 18)=6.812 | 0.02 |  |
| 3-Hydroxysebacic Acid | Y Maze | 0.2496 | -0.4996 | F(1, 18)=5.987 | 0.02 | NeuN+FJB | 0.4132 | 0.6428 | F(1, 18)=12.68 | 0.002 |  |
|  | Rotarod-Latency | 0.2462 | -0.4962 | F(1, 18)=5.878 | 0.02 |  |  |  |  |  |  |
|  | Rotarod-Speed | 0.22 | -0.4690 | F(1, 18)=5.017 | 0.04 |  |  |  |  |  |  |
| Metabolites detected by targeted metabolomics | | | | | | | | | | | |
| LTB4 |  |  |  |  |  | NeuN+TUNEL | 0.2651 | -0.5149 | F(1, 18)= 6.494 | 0.0202 | Arachidonic acid metabolism |
|  |  |  |  |  |  | NeuN+FJB | 0.1652 | -0.4064 | F(1, 18)= 3.561 | <0.0754 | Biosynthesis of unsaturated fatty acids |
| 14,15-DHET | Y Maze | 0.2355 | 0.4853 | F(1, 18)=5.544 | 0.0301 | HE | 0.3421 | 0.5849 | F(1, 18)=9.36 | 0.0068 | Arachidonic acid metabolism |
|  |  |  |  |  |  | NeuN+FJB | 0.2013 | 0.4487 | F(1, 18)=4.536 | 0.0473 | Biosynthesis of unsaturated fatty acids |
| 5(S)-HETE |  |  |  |  |  | HE | 0.2158 | -0.4645 | F(1, 18)=4.954 | 0.0390 | Arachidonic acid metabolism; |
|  |  |  |  |  |  | NeuN+FJB | 0.3170 | -0.5630 | F(1, 18)=8.355 | 0.0095 | Biosynthesis of unsaturated fatty acids |
| 5(S)-HPETE | RadialMaze-Time | 0.2133 | 0.4618 | F(1, 18)=4.879 | 0.0404 | HE | 0.3759 | 0.6131 | F(1, 18)=10.84 | 0.0040 | Arachidonic acid metabolism; |
|  | RotaRos-Speed | 0.2215 | -0.4701 | F(1, 18)=5.123 | 0.0362 | NeuN+TUNEL | 0.2554 | 0.5154 | F(1, 18)=6.174 | 0.0230 | Biosynthesis of unsaturated fatty acids |
| Prostaglandin B2 (PGB2) |  |  |  |  |  | ThioS | 0.2394 | -0.4893 | F(1, 18)=5.664 | 0.0286 | Arachidonic acid metabolism; |
|  |  |  |  |  |  | NeuN+TUNEL | 0.3260 | -0.5710 | F(1, 18)=8.706 | 0.0086 | Biosynthesis of unsaturated fatty acids |
| Dihomo-γ-linolenic acid (DGLA) | RadialMaze-Time | 0.2379 | -0.4878 | F(1, 18)=5.620 | 0.0291 | NeuN+TUNEL | 0.2868 | -0.5355 | F(1, 18)=7.237 | 0.0150 | Linoleic acid metabolism  Biosynthesis of unsaturated fatty acids |
|  |  |  |  |  |  | NeuN+FJB | 0.2636 | -0.5134 | F(1, 18)=6.443 | 0.0206 |  |
| Dehydroepiandrosterone (DHEA) | Y Maze | 0.2757 | -0.5251 | F(1, 18)=6.853 | 0.0174 | HE | 0.2067 | 0.4546 | F(1, 18)=4.689 | 0.044 | Steroid hormone biosynthesis |
|  |  |  |  |  |  | ThioS | 0.2606 | 0.5105 | F(1, 18)=6.343 | 0.0215 |  |
|  |  |  |  |  |  | NeuN+TUNEL | 0.2135 | 0.4621 | F(1, 18)=4.887 | 0.0403 |  |

Metabolites not listed indicate no correlation (|R|<0.4) with behavioral and histological tests
